# Emergence of comparable covalency in isostructural cerium(iv)– and uranium(iv)–carbon multiple bonds†
†Electronic supplementary information (ESI) available: Experimental considerations, crystallographic, and computational details for compounds **2** and **3**. CCDC 938905 and 938904. For ESI and crystallographic data in CIF or other electronic format see DOI: 10.1039/c6sc00278a


**DOI:** 10.1039/c6sc00278a

**Published:** 2016-02-04

**Authors:** Matthew Gregson, Erli Lu, Floriana Tuna, Eric J. L. McInnes, Christoph Hennig, Andreas C. Scheinost, Jonathan McMaster, William Lewis, Alexander J. Blake, Andrew Kerridge, Stephen T. Liddle

**Affiliations:** a School of Chemistry , The University of Manchester , Oxford Road , Manchester , M13 9PL , UK . Email: steve.liddle@manchester.ac.uk; b EPSRC National UK EPR Facility , School of Chemistry and Photon Science Institute , The University of Manchester , Oxford Road , Manchester , M13 9PL , UK; c Helmholtz-Zentrum Dresden-Rossendorf , Institute of Resource Ecology , Bautzner Landstrasse 400 , D-01314 Dresden , Germany; d The Rossendorf Beamline , ESRF , BP 220 , F-38043 Grenoble , France; e School of Chemistry , University of Nottingham , University Park , Nottingham , NG7 2RD , UK; f Department of Chemistry , Lancaster University , Lancaster , LA1 4YB , UK . Email: a.kerridge@lancaster.ac.uk

## Abstract

We report comparable levels of covalency in cerium– and uranium–carbon multiple bonds in the iso-structural carbene complexes [M(BIPM^TMS^)(ODipp)_2_] [M = Ce (**1**), U (**2**), Th (**3**); BIPM^TMS^ = C(PPh_2_NSiMe_3_)_2_; Dipp = C_6_H_3_-2,6-^i^Pr_2_] whereas for M = Th the M=C bond interaction is much more ionic. On the basis of single crystal X-ray diffraction, NMR, IR, EPR, and XANES spectroscopies, and SQUID magnetometry complexes **1–3** are confirmed formally as *bona fide* metal(iv) complexes. In order to avoid the deficiencies of orbital-based theoretical analysis approaches we probed the bonding of **1–3***via* analysis of RASSCF- and CASSCF-derived densities that explicitly treats the orbital energy near-degeneracy and overlap contributions to covalency. For these complexes similar levels of covalency are found for cerium(iv) and uranium(iv), whereas thorium(iv) is found to be more ionic, and this trend is independently found in all computational methods employed. The computationally determined trends in covalency of these systems of Ce ∼ U > Th are also reproduced in experimental exchange reactions of **1–3** with MCl_4_ salts where **1** and **2** do not exchange with ThCl_4_, but **3** does exchange with MCl_4_ (M = Ce, U) and **1** and **2** react with UCl_4_ and CeCl_4_, respectively, to establish equilibria. This study therefore provides complementary theoretical and experimental evidence that contrasts to the accepted description that generally lanthanide–ligand bonding in non-zero oxidation state complexes is overwhelmingly ionic but that of uranium is more covalent.

## Abstract 2

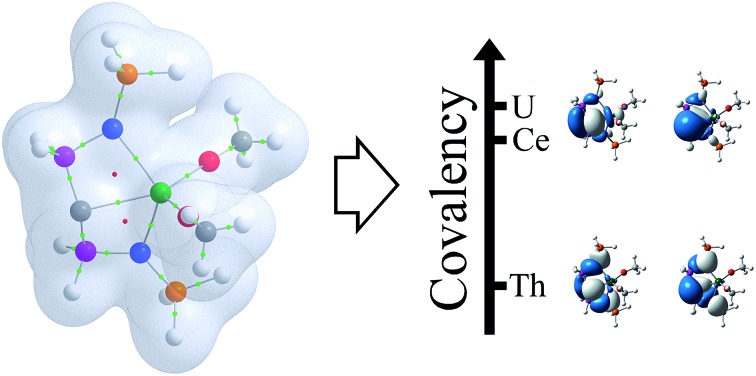
Against expectations the covalency in a cerium(iv)–carbon multiple bond interaction is essentially as covalent as the uranium(iv) analogue.

## Introduction

Ever since the publication of *Nature of the Chemical Bond* over 75 years ago, chemists have vigorously debated the nature of chemical bonding.^1^ Nevertheless, it is instructive to conduct research in the chemical sciences within a guiding framework of general bonding descriptions for different areas of the periodic table. Models have suggested variable levels of covalency for transition and early actinide metals whereas the lanthanides and late actinides are generally regarded as being essentially ionic like alkali and alkaline earth metals.^2^ However, this status quo, especially in f-block chemistry, is continuously being challenged,^3^ as advances in synthesis and characterisation techniques continuously refine our understanding of these elements.^4^

A comparison of chemical bonding that is often made is between 4f cerium and 5f uranium, since according to Shannon their ionic radii are very similar (0.87 *vs.* 0.89 Å and 1.01 *vs.* 1.03 Å for the +IV and +III oxidation states, respectively);^5^ whilst acknowledging that metal oxidation state and the nature of coordinated ligands directly impact the level of covalency in metal–ligand bonding, for the former the valence 4f-orbitals are generally regarded as ‘core-like’ and chemically inaccessible, whereas for the latter the 5f-orbitals are viewed as chemically accessible and able to engage in modest covalent overlap with ligand frontier orbitals.^6^ This view finds support from many reactivity and physical measurements, and, for example, the optical spectroscopy and magnetism of uranium complexes is certainly variable and ligand-field-dependent,^7^ whereas that of lanthanide(iii) complexes is generally described as being independent of the ligand environment and ‘free-ion-like’.^2^,^8^ However, although scattered throughout the literature there are hints that this appealing yet simple description may be misleading. As an example, for cerium(iii) the [Xe]4f^1^ → [Xe]4f^0^5d^1^ transition is found to depend strongly on the ligand field, varying from 49 737 cm^–1^ for gaseous Ce^3+^, to 22 000 cm^–1^ for Ce^3+^ doped into Y_3_Al_5_O_12_, to 17 650 cm^–1^ for [Ce{η^5^-C_5_H_3_(SiMe_3_)_2_}_3_].^9^ Furthermore, a number of studies have suggested that the presence of covalent bonding in 4f complexes should be seriously considered.4f,^10^ An additional point, is that thorium, although exhibiting a larger ionic radius than uranium or cerium (0.94 Å),^5^ resides like cerium at the start of the f-block and so it is of interest to determine similarities or differences in the chemical bonding of these 4f *vs.* 5f elements. Overall, for an isostructural pair of tetravalent uranium and cerium complexes, the order of covalency involving those metal centres would normally be expected to be uranium significantly greater than cerium. This is important to understand, from a fundamental perspective, but there are also practical implications; these three elements can be found in the presence of one another in spent nuclear fuel and future strategies to separate them might depend on exploiting differences in their covalent chemical bonding.^11^ Since f-elements have existing and increasing industrial roles in catalysis, magnets, photonics, alloys, energy, and national security it is increasingly desirable to garner a better understanding of the electronic structure and chemical bonding of these elements.

Despite many studies of uranium, cerium, and thorium complexes, comparative studies of the covalency in their chemical bonding are quite rare, and where documented when this study was initiated usually reinforced standard descriptions,^12^,^13^ though there is not a consensus.^14^ Multi-configurational calculations on uranocene, thorocene, and cerocene return bonding descriptions that order the covalency as uranium > thorium > cerium,^12^ and studies of M–L (M = U, Ce; L = σ-donor ligand) all suggest the bonding of uranium to be much more covalent than cerium.^13^ Furthermore, in lanthanide complexes demonstrating some degree of covalent character, calculations have suggested that 5d, 6s and 6p orbitals play a more prominent role in metal–ligand bonding than the 4f.^15^ At this point, what is to be defined as covalency merits discussion. The mixing coefficient is proportional to the spatial overlap of the orbitals divided by the difference in their energies and the spatial overlap and energy separations are independent parameters.^16^ Thus, increased covalency may be associated with increased spatial overlap or increased orbital energy near-degeneracy. Although the latter definition is certainly valid, whether it constitutes covalency in the generally chemically accepted view is an interesting question, since covalent chemical bonding carries the connotation of overlap resulting in a build-up of electron density in the inter-nuclear region. It is worth noting at this point that orbital energy levels are not well-defined for all quantum-chemical methodologies, and so probing covalency with an orbital-based computational methodology may not be appropriate. Therefore, this study focuses on an electron density approach rather than orbital structure. This is appropriate in the context of covalency described by spatial overlap; indeed, Pauling referred to covalent bonds as “*the sharing of a pair of electrons by the two bonded atoms*”.^1^ This approach permits us to probe exactly this electron sharing in an orthogonal and complementary manner to methods such as XANES ligand *K*-edge spectroscopy that probe transitions to unoccupied orbitals and extrapolates from this covalency defined on the basis of orbital energy near-degeneracy.^4^

Recently, as part of a wider effort to prepare lanthanide–carbon multiple bonds,^17^ we reported the well-defined cerium(iv) carbene diaryloxide complex [Ce(BIPM^TMS^)(ODipp)_2_] [**1**, BIPM^TMS^ = C(PPh_2_NSiMe_3_)_2_; Dipp = C_6_H_3_-2,6-^i^Pr_2_].^18^ Complex **1** is notable for being a cerium(iv) organometallic and containing a Ce=C multiple bonding interaction. Whilst dominated by electrostatics, this bond exhibits covalency according to NBO analysis of DFT-derived densities. There is reason to have confidence in such analysis as SAOP/ZORA/TZP TD-DFT calculations at the same level of theory reproduce very well the experimentally observed UV/Vis/NIR spectrum. NBO analysis identifies ∼13% cerium character in each of two Ce–C bonding interactions (σ + π). Non-aqueous cerium(iv)^19^ is often a difficult oxidation state to access in an organometallic arena,13c,^18^,^20^ and the 4^th^ ionisation energy of cerium is greater than the sum of the first three;^21^ however, with **1** in-hand, we surmised that as uranium(iv) and thorium(iv) are robust oxidation states, the synthesis of **1** presents an opportunity to directly compare the nature of the chemical bonding of cerium, uranium, and thorium. Here, we report the synthesis and characterisation of [M(BIPM^TMS^)(ODipp)_2_] (M = U, **2**; Th, **3**); the synthesis of **2** and **3** are straightforward, but importantly permit a comparison of the bonding of three isostructural complexes. Surprisingly, both DFT (*via* both orbital- and density-based analyses) and CASSCF/RASSCF (*via* density-based analysis) methods suggest that the covalency and f-orbital interactions for the cerium and uranium complexes are essentially the same, in contrast to the thorium complex that is essentially ionic. The emergence of these results is in contrast to almost all other examples of comparative studies of 4f and 5f covalency,^12^,^13^ and suggests that the established purely ionic general bonding picture of lanthanide cations does not always hold true. Interestingly, this has also recently been suggested by an orthogonal XANES spectroscopy study reported during this work that probed simple cerium(iv) and uranium(iv) hexachloride dianion salts, where on the basis of orbital energy near-degeneracy similar levels of covalency between cerium(iv) and uranium(iv) have been proposed.4*f* The theoretical description of the relative levels of covalency in **1–3** are also consistent with experimental exchange reactions with metal tetrahalide salts of cerium, uranium, and thorium, further supporting our findings.

## Results and discussion

### Synthesis and characterisation

**Scheme 1:**
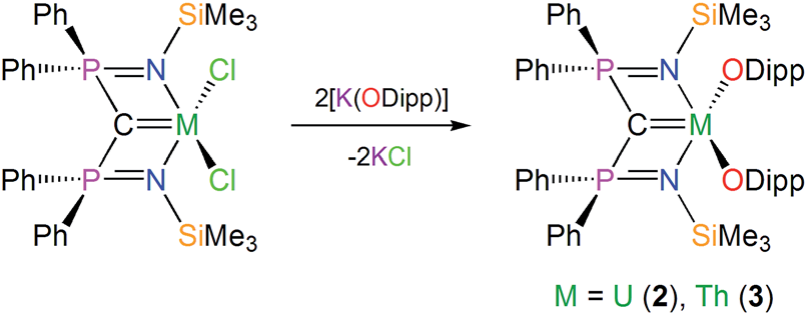
Generic, representative syntheses of **2** and **3**. For the synthesis of **1** see ref. 18.

In contrast to **1**, which required a multi-step preparation,^18^ the synthesis of **2** and **3** was straightforwardly accomplished by installation of the BIPM^TMS^ carbene then the two aryloxides onto uranium or thorium by sequential salt elimination reactions, Scheme 1. After work-up, complexes **2** and **3** were isolated as brown and colourless crystals in 56 and 61% yield, respectively. The ^1^H NMR spectrum of **2** spans the range –19 to +17 ppm and the ^31^P NMR spectrum exhibits a broad resonance at –293 ppm, consistent with the uranium(iv) formulation that is supported by a solution magnetic moment of 2.75 *μ*_B_ at 298 K. In contrast, the ^1^H NMR spectrum of **3** spans the range 0 to +8 ppm and the ^31^P NMR resonance appears at +4.7 ppm. The electronic absorption spectrum of **2** (see ESI†) is characterised by weak (*ε* < 80 M^–1^ cm^–1^) absorptions over the range 500–1900 nm that are characteristic of Laporte forbidden f–f transitions for the ^3^H_4_ electronic manifold of the 5f^2^ uranium ion^22^ whereas for **3** the spectrum is featureless over 400–2000 nm as expected for its colourless 6d^0^5f^0^ nature. As reported previously, the electronic absorption spectrum of **1** exhibits two broad absorptions in the visible region (435 and 541 nm; *ε* = 4560 and 5365 M^–1^ cm^–1^, respectively), the broadness and resulting purple colour of which is a defining feature of many cerium(iv) complexes.^23^

### Solid state structures

**Fig. 1:**
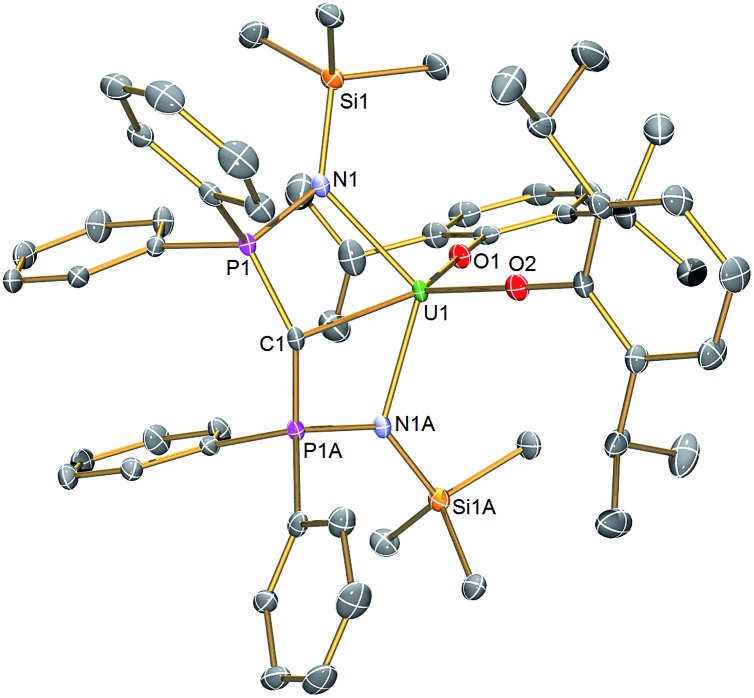
Molecular structure of **2**. Displacement ellipsoids set to 40% and hydrogen atoms omitted for clarity. Complexes **1** and **3** are isostructural and therefore essentially identical in appearance. Selected bond lengths (Å) for **1**: Ce1–C1 2.441(5), Ce1–N1 2.374(3), Ce1–N1A 2.374(3), Ce1–O1 2.137(4), Ce1–O2 2.130(4), C1–P1 1.692(2), C1–P1A 1.692(2), P1–N1 1.626(3), P1A–N1A 1.626(3). For **2**: U1–C1 2.414(3), U1–N1 2.349(2), U1–N1A 2.349(2), U1–O1 2.124(2), U1–O2 2.144(2), C1–P1 1.681(2), C1–P1A 1.681(2), P1–N1 1.640(2), P1A–N1A 1.640(2). For **3**: Th1–C1 2.508(5), Th1–N1 2.416(3), Th1–N1A 2.416(3), Th1–O1 2.187(4), Th1–O2 2.205(4), C1–P1 1.670(2), C1–P1A 1.670(2), P1–N1 1.640(3), P1A–N1A 1.640(3).

The molecular structures of **2** and **3** were determined by single crystal X-ray crystallography, Fig. 1 (see ESI†). The salient feature of isostructural **1–3** is a monomeric formulation with terminal M=C bonds. The remaining coordination sphere of each metal is completed by two BIPM^TMS^ imino chelate arms and the two aryloxide oxygen centres which enforce a pseudo square-based pyramidal geometry. We found a Ce=C distance of 2.441(5) Å in **1**;^18^ this is longer than the Ce=C bonds reported in the theoretical models of CeCH_2_^+^ and Cp_2_CeCH_2_ complexes,15a,^24^ but CeCH_2_^+^ and Cp_2_CeCH_2_ are experimentally unknown, sterically unimpeded and, in the case of the former, benefit from the reduced electronic repulsion associated with a net positive charge. For experimentally realised compounds, the Ce=C distance of **1** is amongst the shortest ever reported, except for the special case of fullerene encapsulated Ce_2_.^25^ The U=C and Th=C distances in **2** and **3** were determined to be 2.414(3) and 2.508(5) Å, respectively; on the basis of Shannon's ionic radii^5^ the former is ∼0.05 Å shorter than would be anticipated but the latter is as would be expected and both are consistent with U=C and Th=C bonds in BIPM^TMS^ complexes.^26^

### Magnetism and electron paramagnetic resonance spectroscopy studies

**Fig. 2:**
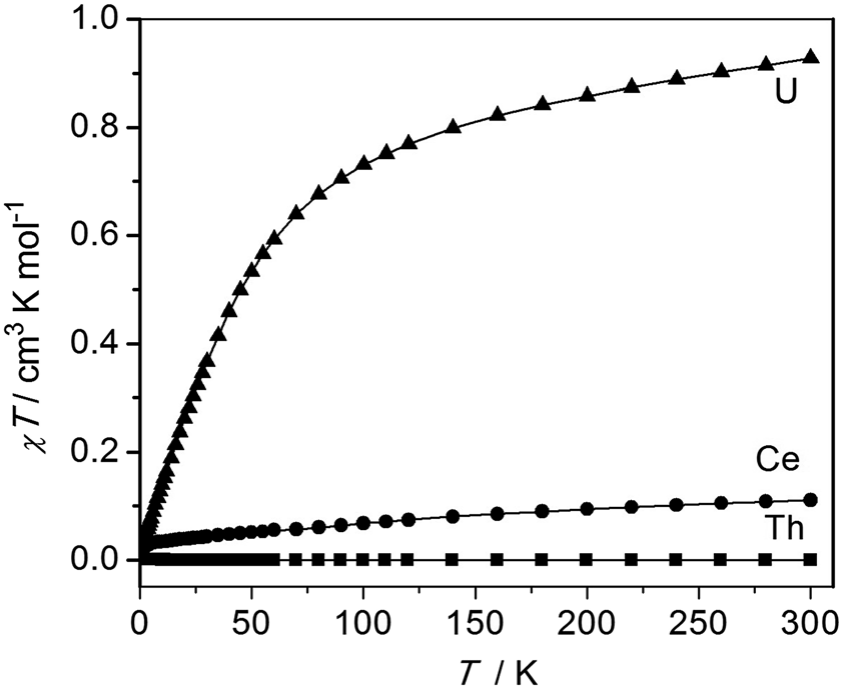
Magnetic data of solid samples of **1–3**, measured in 5000 G applied magnetic field. Diamagnetic corrections for **1** and **2** were estimated from the data for **3**; the weak paramagnetism from samples of **1** is due to an impurity (see ESI†).

The oxidation state assignments of **1–3** are also consistent with solid state magnetic measurements, Fig. 2 (and see ESI†). Complex **2** has a room temperature *χT* of 0.93 cm^3^ K mol^–1^ (equivalent to a magnetic moment 2.73 *μ*_B_, in agreement with solution studies; *χ* is the molar magnetic susceptibility) and decreases rapidly on cooling tending to zero, which is typical for 5f^2^ uranium(iv) that is a magnetic singlet at low temperature.^6^,^27^ Complex **3** is diamagnetic, consistent with closed-shell thorium(iv). Studies of **1** give a very small magnetic moment (0.02–0.1 cm^3^ K mol^–1^ depending on temperature and diamagnetic corrections) that varies from batch to batch (see ESI†). Magnetic data for the cerium and uranium samples **1** and **2** were corrected for diamagnetic contributions by subtraction of data of the Th complex **2**. However, this procedure is not exact, for example not perfectly accounting for the sealed pyrex sample tubes (which have small magnetic impurities) nor the difference between the 4f (Ce) and 5f (Th) ion diamagnetism. Because measured samples of **1** have a very weak paramagnetism, the precise nature of the *χT*(*T*) plot (the absolute value, particularly at high temperature, and the shallow slope in Fig. 2) is very sensitive to the correction applied and should not be over-interpreted. There is no such problem for the strongly paramagnetic **2**.

The weak paramagnetic response from samples of **1** is only consistent with a small quantity of paramagnetic impurity where the bulk of the sample is diamagnetic, consistent with cerium(iv) or other diamagnetic configurations (admixtures of cerium(iv) with singlet cerium(iii) + radical ligand configurations have been proposed for cerocene).20*f* The measured paramagnetism is only *ca.* 5–15% of values expected for f^1^ cerium(iii) (0.8 cm^3^ K mol^–1^ calculated for ^2^F_5/2_; measured values for anionic cerocene derivatives are 0.6–0.7 cm^3^ K mol^–1^ at room temperature decreasing to 0.4–0.6 cm^3^ K mol^–1^ at 2 K).20f,^28^ That the highly air-sensitive **1** decomposes to give cerium(iii) – we note the related complex [Ce(BIPM^TMS^)(ODipp)(THF)]^18^ is very unstable and decomposes surprisingly easily – is also consistent with low-temperature X-band EPR spectra which have (batch-dependent) broad features at *g*_eff,∥_ = 3.7 and *g*_eff,⊥_ = 0.85 (see ESI†). That this is an impurity signal is confirmed by the fact that these *g*_eff_ values would give a magnetic moment of *ca.* 0.5 cm^3^ K mol^–1^ if they derived from the bulk species. Both **2** and **3** are EPR silent, as expected. Although the magnetic and EPR data for **1** are consistent with only the presence of magnetic impurities, they do not, because of the diamagnetic correction being large compared to the weak paramagnetism, rule out excited state mixing, *i.e.* a multi-configurational ground state, due to the shallow positive gradient that could be attributed to temperature independent paramagnetism. In order to conclusively show that **1** is cerium(iv), and therefore that comparisons to **2** and **3** are valid, we recorded the XANES spectrum of **1**.

### X-ray absorption near edge structure spectroscopy

**Fig. 3:**
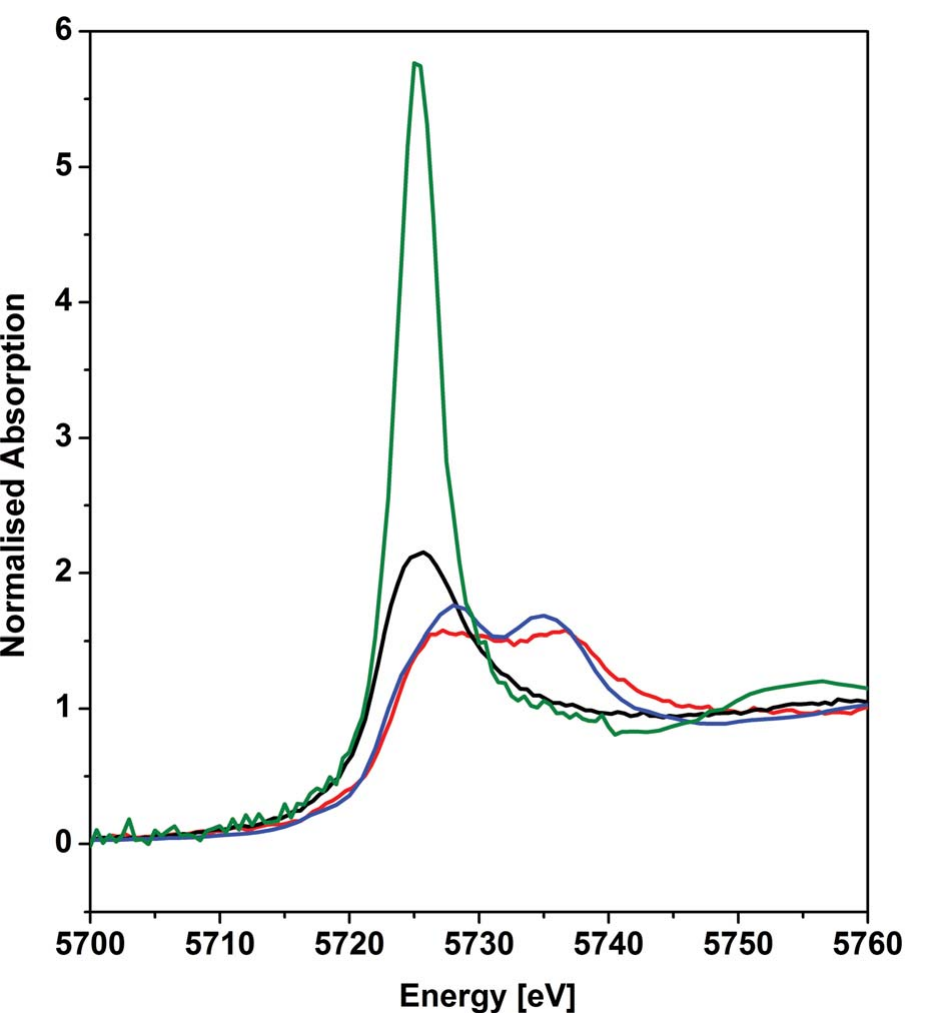
Cerium L_III_-edge XANES spectrum of the cerium(iv) complex **1** (red trace) in comparison to its cerium(iii) precursor [Ce(BIPM^TMS^)(ODipp)_2_K(THF)] (black trace). As references, spectra of 0.01 M cerium(iii) nitrate in water (green trace), and of cerium(iv) dioxide (blue trace) are given. The XANES spectra of **1** and its precursor were recorded at 15 K and the references were recorded at 298 K.

The cerium L_III_-edge spectrum of 0.01 M Ce(NO_3_)_3_ in water, the cerium(iii) precursor to **1** [Ce(BIPM^TMS^)(ODipp)_2_K(THF)], CeO_2_, and **1** are illustrated in Fig. 3. As expected for cerium(iii) complexes, the L_III_-edge spectra of aqueous Ce(NO_3_)_3_ and [Ce(BIPM^TMS^)(ODipp)_2_K(THF)] both consist of a single peak, just above the absorption threshold, at ∼5725.7 eV that is characteristic of cerium(iii).^29^ In contrast, the L_III_-edge spectra of CeO_2_ and **1** both exhibit the characteristic double absorption features of cerium(iv) at ∼5727.2 and ∼5736.7 eV, that are similar to those of CeF_4_, Ce(SO_4_)_2_·4H_2_O, and CeCl_6_^2–^.4f,^30^ The L_III_-edge E_1_ absorption for **1** is ∼1.5 eV higher in energy than the corresponding [Ce(BIPM^TMS^)(ODipp)_2_K(THF)] L_III_-edge absorption, as found generally for cerium(iv) complexes.4f,^29^ The double-peak ratio for CeO_2_ and **1** are both essentially 1 : 1, as has been found for CeCl_6_^2–^ and CeF_4_,4f,^30^ but it is markedly different to that of cerocene.^31^,^32^ The double absorptions could be interpreted in different ways, either as a 4f^1^ L^–1^ contribution to a multiconfigurational ground state,^33^ or resulting from final state effects, *i.e.* a multiconfigurational *excited* state.4f,^34^ In this regard, opinion in the literature is divided and the topic is intensively debated, but it is interesting to note that systems with relatively innocent ligands such as chloride and oxide give spectra with features that are energetically similar to more electronically complex molecules such as cerocene, but with different double-peak ratios. Variable-pressure and theoretical studies have both suggested that the double-absorption spectrum of cerium(iv) complexes with oxide and halide ligands is due to final state effects,4f,30d whereas for cerocene this has been attributed to multiconfigurational ground state effects.^31^ In that regard, the XANES spectrum of complex **1** is certainly much more like that of CeO_2_, CeF_4_, Ce(SO_4_)_2_·4H_2_O, and CeCl_6_^2–^ than cerocene; this observation is consistent with the premise that an open-shell singlet or triplet formulation of **1** should be regarded as less likely than a closed-shell singlet and so we conclude that the presence of cerium(iii) character in **1** can be excluded.

### Theoretical characterisation

Since the XANES data suggest that **1** possesses formal cerium(iv) character, it can legitimately be compared to **2** and **3**. We previously reported Natural Bond Orbital (NBO) data for **1** which returned σ- and π-bonds composed of ∼13% Ce and ∼87% C character.^18^ In the σ-bond cerium principally employs 4f (76%) and 5d (21%) character whereas the π-bond exhibits high 4f (80%) and 5d (19%) contributions. When interrogating **2** by NBO, a similar breakdown is returned. Specifically, the σ-bond is composed of 16% U and 84% C and the π-bond is made up of 14% U and 86% C character. The U contributions to the σ- and π-bonds are 5f (87%) and 6d (12%), and 5f (77%) and 6d (22%), respectively. In contrast, the NBO data for **3** return ionic interactions with localised carbene lone pairs with no Th character as the contribution of the latter falls below the default cut-off of 5% in the NBO code. These calculations suggest that, whilst the bonding between cerium–, uranium–, and thorium–carbon centres in **1–3** are dominated by ionic interactions, a modest and surprisingly comparable covalent contribution to the bonding is evident in **1** and **2** despite the commonly held view that lanthanide–ligand chemical bonding is purely ionic.

Although the NBO calculations are internally consistent and well suited to describing covalency in molecules,^35^ they are based on results from DFT calculations that have well-documented shortcomings with respect to the treatment of electron correlation. Thus, we turned to multi-configurational calculations to develop a quantitative, meaningful description of the chemical bonding of the M=C units in **1–3**. These calculations employed the restricted-active-space self-consistent field (RASSCF) theory,^36^ which completely avoids the problems inherent to DFT studies of open-shell systems by treating static electron correlation explicitly *via* a configuration interaction approach. Whilst RASSCF is a powerful technique for elucidating the nature of metal–ligand interactions in complexes such as those considered here, it is limited in the size of systems to which it can be applied. For this reason, complexes **1–3** were truncated in order to render RASSCF calculations computationally tractable by replacing *P*-phenyls with H, silyl-methyls with H, and the bulky Dipp groups by Me. This truncation retains the coordination environment of all atoms directly bonded to the metal: where hydrogen termination was employed, only the positions of the terminating hydrogens were optimised.

In order to assess any truncation effects on the electronic structures, ground state electron densities were calculated at the PBE/TZVP level of theory. These densities were probed with the quantum theory of atoms in molecules (QTAIM) approach^37^ since, in contrast to orbital-based measures, multi-configurational studies of cerium(iv) complexes have shown density-based analysis methods provide unambiguous electronic structure interpretations.^12^,^18^,^38^ Furthermore, this density-based approach allows us to consider all contributions to covalent bonding character, irrespective of the orbital origin. We focus on two key properties: the delocalisation index (*δ*), a quantitative measure of the degree of electron sharing between two atomic centres,^39^ and the magnitude of the electron density at the M=C bond critical point (*ρ*), an accepted measure of covalency. These two measures, while complementary, are not equivalent: *ρ* provides a quantitative measure of charge accumulation in the bonding region, which is related to spatial overlap, whereas the delocalisation index, *δ*, between two bonded atoms is maximised when electrons are shared equally which, in a monodeterminantal framework, is a manifestation of orbital degeneracy. This analysis therefore allows us to determine the variation in both of these phenomena when the metal centre is varied and has previously been reported in several studies of cerium and uranium complexes.^12^,^13^,^38^,^40^ Reassuringly, reductions of <2% in *ρ* and <3% in *δ* are observed when comparing full and truncated complexes, demonstrating that the quantitative bonding characteristics of the full complexes **1–3** is retained.

The electronic structures of the truncated complexes were then evaluated using the RASSCF methodology. These calculations employed three active spaces: RAS1, containing only occupied orbitals from the monodeterminantal reference wavefunction, RAS2, containing both occupied and virtual orbitals, and RAS3, containing only virtual orbitals. Full configuration interaction (CI) was performed in RAS2, while truncated CI, considering only singly and doubly excited configurations, was performed between the RAS1, RAS2 and RAS3 subspaces. All active space orbitals were optimised. Due to the large computational costs of such calculations, the RAS1, RAS2 and RAS3 subspaces were restricted to 11, 7 and 11 orbitals, respectively: the 7 RAS2 orbitals incorporated the 4f/5f manifold, whereas the RAS1 and RAS3 subspaces included all orbitals with significant carbon and nitrogen 2s and 2p character. The oxygen 2s and 2p orbitals could not be included in the active subspaces, since attempts resulted in the intrusion of phosphorus-based orbitals. It was observed, however, that oxygen 2s and 2p orbitals that were successfully stabilised in the active subspaces exhibited occupation numbers extremely close to integer values. Similarly, occupation numbers of formally unoccupied d-orbitals was effectively 0, indicating that the inclusion of these orbitals in the active subspaces is not required. It may be that the geometric constraints of BIPM^TMS^ favour f- over d-orbital participation in the bonding of this ligand to f-elements generally, but further studies will be required to confirm this. This definition of the active subspaces resulted in RASSCF(*n*,2,2;11,7,11) calculations. The number of explicitly correlated electrons, *n*, was 22 for complexes **1** and **3** and 24 for complex **2**. In all cases, calculations were performed in *C*_s_ symmetry.

The results of these calculations reveal that all complexes are dominated by electronic configurations corresponding to metal(iv) centres, in agreement with our experimental measurements, and these configurations contribute 89.0, 89.5 and 89.3% to the ground state RASSCF wavefunctions of **1–3** (which are of ^1^*A*′, ^3^*A*′′, and ^1^*A*′ symmetry), respectively. Maximum deviations from integer values in natural orbital occupations were 0.032, 0.033, and 0.025, respectively, indicating rather weak multi-configurational character.^41^ The lack of strong multi-configurational character in the cerium complex, supported by experimental data, is in stark contrast to that found in cerocene.12a,20d,20f,38a,^42^ For all complexes under consideration, it was found that only the natural orbitals of σ and π M–C (anti-)bonding character exhibited significant deviation from integer occupation, indicating that a simplified complete active space (CAS) comprising 4 electrons correlated in 4 orbitals (or 6 electrons in 6 orbitals to incorporate the 5f^2^ configuration of the uranium compound) should be sufficient to accurately describe the M=C bonding interaction. Subsequent analysis of CASSCF-derived densities revealed them to be extremely similar to their RASSCF counterparts (see Tables S15 and S16 of the ESI†). In the following discussion, however, all quantities are derived from RASSCF calculations.

**Fig. 4:**
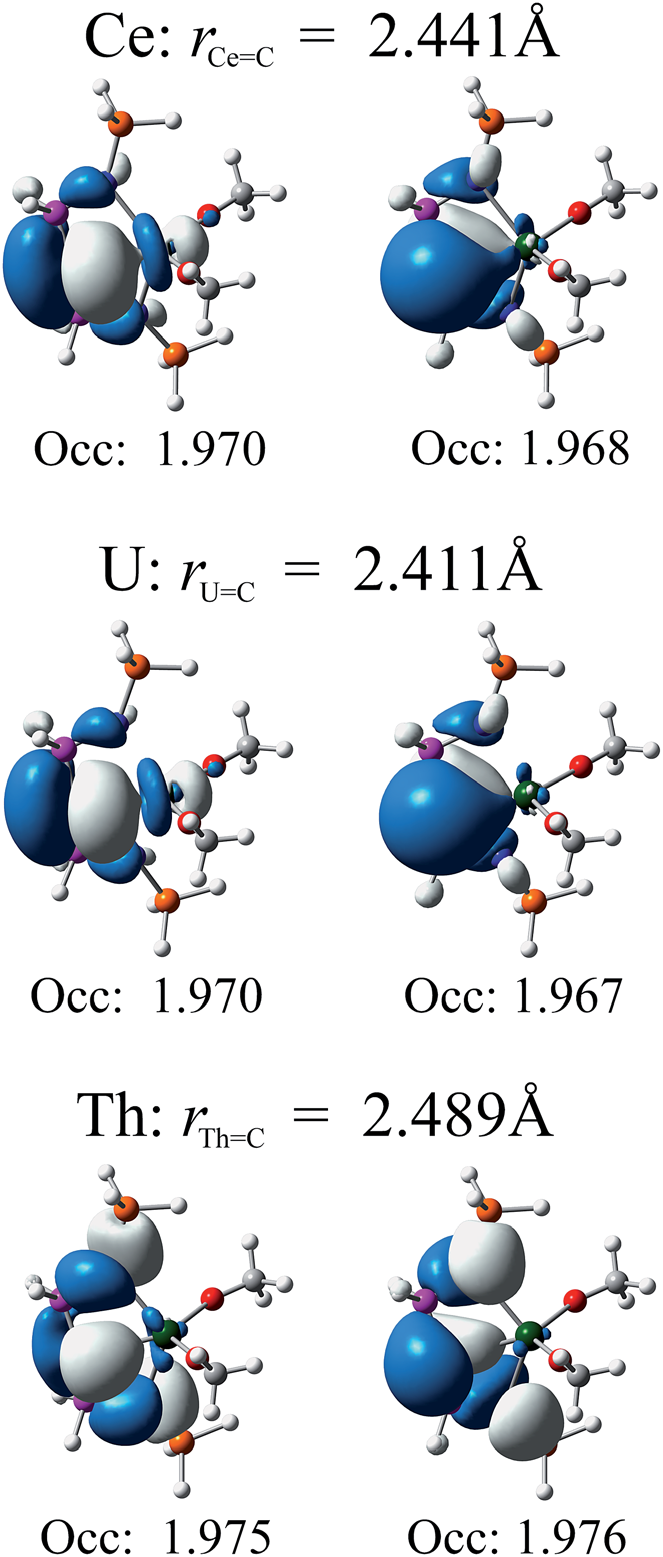
Relevant natural orbitals and corresponding occupancies obtained from RASSCF calculations on the truncated complexes. All orbitals rendered using an isosurface value of 0.04.

In Fig. 4 we present relevant natural orbitals for each complex. The similarity of these orbitals in the cerium and uranium complexes, as well as the near-identical occupation numbers, is startling. In both cases clear σ- and π-bonding character can be seen, in contrast to the ligand-localised orbitals in the thorium complex. The two singly-occupied 5f orbitals in the uranium complex are almost entirely localised on the uranium centre (∼98% 5f character), with negligible ligand contributions.

The RASSCF-calculated wavefunctions were used to obtain explicitly correlated electron densities for subsequent QTAIM analysis. Metal charges are all significantly higher than those found using DFT, increasing by 0.68, 0.59 and 0.49 to give absolute values of +2.84, +2.89 and +3.02 a.u. for **1–3**, respectively, and result in very similar cerium and uranium charges which are notably lower than the thorium charge, indicating greater ionic character in the latter. An increase in *ρ* is also found, but is less pronounced: 0.0051, 0.0020 and 0.0028 a.u., to give absolute values of 0.0784, 0.0859 and 0.0756 a.u., respectively. The increase is most pronounced in the cerium complex, and results in covalent character larger than that found in the thorium analogue. Whilst an overall reduction in *δ* is found, –0.238, –0.229, –0.120 a.u. to give absolute values of 0.538, 0.543 and 0.493 a.u. for **1–3**, respectively, the resulting values also indicate higher covalency in the cerium complex when compared to the thorium analogue and, indeed, demonstrate covalent character of almost the same magnitude as that found in the uranium complex. When combined, these data provide strong evidence for ordering the extent of covalency in these complexes as uranium ≈ cerium > thorium in these complexes and thus confirm the premise suggested by the DFT calculations, in stark contrast to previous studies. For example, in the cerium(iv) N-heterocyclic carbene complex [Ce(L){N(SiMe_3_)_2_}_2_F] [L = OCMe_2_CH_2_(CNCH_2_CH_2_N-Dipp)] a *ρ*(Ce,C) of 0.045 a.u. is found,13*c* which is 81.8% of the value found in the analogous uranium complex. Chloro analogues were also considered and *ρ*(Ce,C) was found to be 84.9% of the *ρ*(U,C) value. Similarly, calculations on cerocene^11^ found *ρ*(Ce,C) to be 0.0395, which is 83.0% of the analogous value calculated for uranocene.12*a* Here, we find *ρ*(Ce,C) for **1** to be 91.3% of the corresponding U value in **2**. Comparison of *δ*(Ce,C) can also be made with that in cerocene, where it was found to be 83.2% of the *δ*(U,C) value in uranocene; here, we calculate *δ*(Ce,C) for **1** to be 99.1% of the corresponding *δ*(U,C) value in **2**.

### Exchange reactions and thermodynamic considerations

In order to experimentally probe the relative levels of covalency in **1–3** and calibrate the above calculations, we investigated the exchange reaction chemistry of **1–3** since it is well known that covalency can drive exchange reactions. For example, rare earth tris-cyclopentadienyl complexes readily react with iron halides to afford ferrocene and rare earth halides and this reactivity, whilst undoubtedly reflecting the favourable formation of lanthanide–halide bonds, is driven by the formation of highly covalent iron–cyclopentadienyl bonding.^43^

1 (**1**) + [ThCl_4_(THF)_3.5_] → no reaction

2 (**3**) + [CeCl_4_(HMPA)_2_] → reaction

Apart from the onset of decomposition of **1**, which is known to be unstable in solution, no reaction between **1** and [ThCl_4_(THF)_3.5_] is observed in benzene after a 24 hour stir (eqn (1)). After a 5 day stir **1** is completely decomposed to yield a species that exhibits a resonance at –34 ppm in the ^31^P NMR spectrum. Although we have not been able to isolate and identify this species its ^31^P NMR chemical shift is in the region where related cerium(iii) BIPM^TMS^ complexes exhibit ^31^P NMR resonances.^18^ It would therefore seem that **1** does not react with thorium tetrachloride and instead decomposes before any reactivity can occur. In the reverse scenario, eqn (2), treatment of **3** with [CeCl_4_(HMPA)] [HMPA = OP(NMe_2_)_3_] results in the loss of ^1^H NMR resonances attributable to **3** and evolution of the characteristic purple colour of **1** in the first 15 minutes. After 15 minutes the purple colour fades and an intractable mixture of products is formed. Given that the preparation of **1** is not straightforward it is not surprising that if formed under these less than optimal conditions it would decompose given its instability in solution, but the purple colour is certainly consistent with the exchange of BIPM^TMS^ from thorium(iv) to cerium(iv) and in-line with the proposed differences in covalency.

3 (**2**) + [ThCl_4_(THF)_3.5_] → no reaction

4 (**3**) + [UCl_4_(THF)_3_] → reaction

As with the absence of reaction between **1** and [ThCl_4_(THF)_3.5_], eqn (1), we find that there is also no reaction of **2** with [ThCl_4_(THF)_3.5_], eqn (3). In the reverse situation, eqn (4), **3** does react with [UCl_4_(THF)_3_]. Unfortunately, an intractable product mixture is obtained, likely due to ligand scrambling under conditions that are by definition less controlled than the usual route to prepare **2**. However, it is clear that ^1^H NMR resonances attributable to **3** are lost so the implication is that the BIPM^TMS^ ligand is transferred to uranium. Irrespective of the precise outcomes, these reactions are consistent with uranium being more covalent than thorium.

5 (**1**) + [UCl_4_(THF)_3_] → reaction

6 (**2**) + [CeCl_4_(HMPA)_2_] → reaction

When **1** is treated with [UCl_4_(THF)_3_], eqn (5), the intense purple colour of **1** fades within 30 minutes and is replaced by a green colour which is then replaced by a brown colour consistent with the formation of **2**. In the reverse situation, eqn (6), ^1^H NMR resonances attributable to **2** are lost; no purple colour was observed, but it is clear that ligand exchange has occurred, and we note that after a 5 day stir the mixture exhibits a ^31^P NMR resonance at –34 ppm, which is indicative of a cerium(iii) BIPM^TMS^ derivative.^18^ In order to exclude the possibility that decomposition is due to HMPA we treated **2** with neat HMPA in a control experiment and found that no reaction occurs. The uranium–cerium exchange reactions are not clean, but it is evident that ligand exchange occurs to some extent. Although equilibria are to some extent established, **1** is not stable in solution for extended periods and the evidence suggests that eventually the cerium decomposes to the trivalent state, which then degrades the equilibria.

7 (**1**) + [ThCl_4_(THF)_3_] → (**3**) + [CeCl_4_(THF)_3_], Δ*H*_rxn_ = +11.2 kcal mol^–1^

8 (**3**) + [CeCl_4_(HMPA)_2_] → (**1**) + [ThCl_4_(HMPA)_2_], Δ*H*_rxn_ = –10.2 kcal mol^–1^

9 (**2**) + [ThCl_4_(THF)_3_] → (**3**) + [UCl_4_(THF)_3_], Δ*H*_rxn_ = +13.5 kcal mol^–1^

10 (**3**) + [UCl_4_(THF)_3_] → (**2**) + [ThCl_4_(THF)_3_], Δ*H*_rxn_ = –13.5 kcal mol^–1^

11 (**1**) + [UCl_4_(THF)_3_] → (**2**) + [CeCl_4_(THF)_3_], Δ*H*_rxn_ = –2.3 kcal mol^–1^

12 (**2**) + [CeCl_4_(HMPA)_2_] → (**1**) + [UCl_4_(HMPA)_2_], Δ*H*_rxn_ = +1.8 kcal mol^–1^

To further support the above findings, we determined the theoretical bond enthalpy changes (Δ*H*_rxn_) for the full, balanced versions of eqn (1)–(6), eqn (7)–(12), by calculating the gas phase geometry optimised structures (all-electron BP86/ZORA/TZP level) of all the constituent components. A solvent continuum was not applied since the solvent for eqn (1)–(6) was benzene, which could reasonably be expected to have systematically minimal interactions with the electropositive species in solution. Experimentally, [ThCl_4_(THF)_3.5_] is most likely a separated ion pair formula like related lanthanide triiodides,^44^ so we approximated it to the molecular analogue [ThCl_4_(THF)_3_]. The calculations most likely carry absolute errors of 5–10 kcal mol^–1^, but, assuming that this is to some extent systematic, the relative errors will reduce to ∼2–5 kcal mol^–1^. The calculations are thus clear-cut as they independently and correctly reproduce the experimental outcome in every case.

Overall, these exchange reactions demonstrate that thorium(iv) does not displace BIPM^TMS^ from cerium(iv) or uranium(iv) whereas the latter pair do displace BIPM^TMS^ from the former. When cerium(iv) or uranium(iv) derivatives are mixed it is evident that equilibria are established, but the reactions are not clean and the equilibria are disrupted due to the instability of **1**. Although some of the products of these reactions are not known, the key point is whether a reaction occurs at all or not. The fact that distinct colour changes are observed, or not, suggests that the carbenes are, or not, transferred since it is the M=C bonds that contribute to absorptions in the visible part of the optical spectra for **1** and **2** and the M–ODipp linkages absorb well into the UV-region. Therefore, the conclusion is that thorium(iv) is the most ionic in this context, whereas cerium(iv) and uranium(iv) do exhibit comparable covalency and these observations experimentally support the same theoretical proposition.

## Summary and conclusions

In summary, we have reported the synthesis of **1–3** and on the basis of their characterisation data these complexes can all be described as *bona fide* formal oxidation state iv complexes. This in turn has provided an opportunity to directly compare the degree of covalency in isostructural cerium, uranium, and thorium carbene complexes. We reiterate that while the bonding of the M=C units in these complexes is predominantly ionic, we note a significant covalent contribution to these linkages for cerium and uranium. Significantly, the levels of covalency and f-orbital participation in the M=C bonds are remarkably similar for cerium and uranium, but different from thorium which is ionic. Importantly, the similar levels of covalency in the cerium(iv)– and uranium(iv)–carbon multiple bonds in **1** and **2** manifests in more than one type of theoretical treatment (DFT, RASSCF and CASSCF), and most compellingly is supported by experimental exchange reactions that proceed as predicted from the above covalency arguments. It may be that the similar levels of covalency of cerium(iv) and uranium(iv) is a more general effect than currently recognised, but one that is relatively small and so has eluded detection in systems that exhibit minimal covalency. Since the synthesis of cerium(iv) complexes that go beyond simple salts is still in its infancy, and is experimentally challenging, it may be that more examples of cerium(iv) and uranium(iv) complexes containing similar levels of covalency await discovery. At the very least the results presented here provide a basis to question the established exclusive ionic bonding textbook description of the lanthanides in non-zero oxidation states, especially with reference to certain 5f metals.

## Experimental

### General

All manipulations were carried out using Schlenk techniques, or an MBraun UniLab glovebox, under an atmosphere of dry nitrogen. Solvents were dried by passage through activated alumina towers and degassed before use or were distilled from calcium hydride. All solvents were stored over potassium mirrors, except for ethers that were stored over activated 4 Å sieves. Deuterated solvent was distilled from potassium, degassed by three freeze–pump–thaw cycles and stored under nitrogen. [Ce(BIPM^TMS^)(ODipp)_2_] (**1**),^18^ [K(ODipp)], [U(Cl)_3_(BIPM^TMS^)Li(THF)_2_], and [Th(Cl)_2_(BIPM^TMS^)] were prepared by published methods.26a,b,^45^ ^1^H, ^13^C, ^29^Si, and ^31^P NMR spectra were recorded on a Bruker 400 spectrometer operating at 400.2, 100.6, 79.5, and 162.0 MHz respectively; chemical shifts are quoted in ppm and are relative to TMS (^1^H, ^13^C, ^29^Si) and 85% H_3_PO_4_ (^31^P). FTIR spectra were recorded on a Bruker Tensor 27 spectrometer. UV/Vis/NIR spectra were recorded on a Perkin Elmer Lambda 750 spectrometer. Data were collected in 1 mm path length cuvettes loaded in an MBraun UniLab glovebox and were run *versus* the appropriate reference solvent. Solution magnetic moments were recorded at room temperature using the Evans method. Static variable-temperature magnetic moment data were recorded in an applied dc field of 0.1 T on a Quantum Design MPMS XL7 superconducting quantum interference device (SQUID) magnetometer using doubly recrystallised powdered samples. Care was taken to ensure complete thermalisation of the sample before each data point was measured and samples were immobilised in an eicosane matrix to prevent sample reorientation during measurements. Diamagnetic corrections were applied for using tabulated Pascal constants and measurements were corrected for the effect of the blank sample holders (flame sealed Wilmad NMR tube and straw) and eicosane matrix. Variable temperature (300–5 K) EPR spectra were measured at X-band (*ca.* 9 GHz, respectively) on a Bruker Elexsys E580 spectrometer. Polycrystalline samples were sealed under vacuum in 1 mm i.d. silica tubing, and double-contained for EPR by insertion into an X-band silica tube or PTFE sleeve. CHN microanalyses were carried out by Tong Liu at the University of Nottingham. Cerium L_III_-edge XANES measurements were performed using a Si(111) double-crystal monochromator on the Rossendorf Beamline at the European Synchrotron Radiation Facility (Grenoble, France). Higher harmonics were rejected by two Si coated mirrors. The spectra were collected using ionisation chambers filled with nitrogen and a 13-element Ge fluorescence detector. The samples were measured at 15 K in a closed-cycle He cryostat. The reference samples spectra of 0.01 M Ce(iii) nitrate in H_2_O and solid CeO_2_ were measured at room temperature in transmission mode.

### Preparation of [U(BIPM^TMS^)(ODipp)_2_] (**2**)

THF (15 ml) was added to a precooled (–78 °C) mixture of [U(BIPM^TMS^)(Cl)_3_(Li)(THF)_2_] (1.09 g, 1.0 mmol) and [K(ODipp)] (0.43 g, 2.0 mmol). The resulting brown suspension was allowed to warm to room temperature with stirring over 16 h to afford a brown solution. Volatiles were removed *in vacuo* and the resulting solid was extracted into toluene. Volatiles were removed *in vacuo* to afford a brown solid which upon recrystallisation from Et_2_O (2 ml) at –30 °C afforded **2**·Et_2_O as brown crystals. Yield: 0.69 g, 56%. Anal. calcd for C_59_H_82_N_2_O_3_P_2_Si_2_U: C, 57.91; H, 6.76; N, 2.29%. Found: C, 57.76; H, 6.66; N, 2.33%. ^1^H NMR (C_6_D_6_): *δ* –18.94 (18H, s, NSi(C*H*_3_)_3_), –3.43 (4H, s, C*H*(CH_3_)_2_), –3.25 (24H, s, CH(C*H*_3_)_2_), 1.23 (6H, s, OCH_2_C*H*_3_), 3.34 (4H, s, OC*H*_2_CH_3_), 6.70 (4H, t, Ar-*H*), 8.17 (8H, t, Ar-*H*), 13.40 (2H, t, Dipp-*H*), 16.07 (4H, d, Dipp-*H*), 16.48 (Ar-*H*). ^31^P{^1^H} NMR (C_6_D_6_): *δ* –293.42 (UC*P*_2_). FTIR *ν*/cm^–1^ (Nujol): 1590 (w), 1539 (w), 1403 (w), 1330 (w), 1200 (s), 918 (w), 887 (w), 857 (s), 838 (s), 748 (w), 694 (w), 661 (w). Magnetic moment (Evans method, C_6_D_6_, 298 K): *μ*_eff_ = 2.75 *μ*_B_.

### Preparation of [Th(BIPM^TMS^)(ODipp)_2_] (**3**)

A solution of [Li_2_(BIPM^TMS^)] (0.57 g, 1.0 mmol) in THF (5 ml) was added to a solution of [ThCl_4_(THF)_3.5_] (0.63 g, 1.0 mmol) in THF (5 ml) at –78 °C. The pale yellow mixture was stirred at –78 °C for 30 minutes, then was allowed to warm to room temperature with stirring for 2 h. Volatiles were removed *in vacuo* and DippOK (0.43 g, 2.0 mmol) was added. Toluene (10 ml) was added slowly to the cold (–30 °C) stirring mixture, the resultant mixture was allowed to warm to room temperature with stirring for 1 h. After this time the mixture was filtered, and all volatiles were removed *in vacuo*. The product was recrystallised from a toluene/hexane mixture to yield **3**·0.5(toluene) as colourless crystals. Yield: 0.69 g, 61%. Anal. calcd for C_62_H_80_N_2_O_2_P_2_Si_2_Th: C, 60.27; H, 6.53; N, 2.27%. Found: C, 59.96; H, 6.64; N, 2.45%. ^1^H NMR (C_6_D_6_): *δ* 0.14 (18H, s, NSi(C*H*_3_)_3_), 1.35 (24H, d, ^3^*J*_HH_ = 7.2 Hz, CH(C*H*_3_)_2_), 3.74 (4H, spt, ^3^*J*_HH_ = 7.2 Hz, C*H*(CH_3_)_2_), 6.97–7.04 (14H, m, Ar*H*), 7.21 (4H, d, ^3^*J*_HH_ = 7.6 Hz, Ar*H*), 7.55–7.61 (8H, m, Ar*H*). ^13^C{^1^H} NMR (C_6_D_6_): *δ* 3.18 (s, Si(*C*H_3_)_3_), 24.45 (s, CH(*C*H_3_)_2_), 28.14 (s, *C*HMe_2_), 67.04 (t, *J*_PC_ = 166.7 Hz, Th= *C*P_2_), 120.15, 123.26, 125.66, 128.53, 129.29, 130.11 (Ar*C*), 131.36 (d, ^3^*J*_PC_ = 6.4 Hz, *C*_*meta*_ of P–Ph), 131.43 (d, ^3^*J*_PC_ = 5.5 Hz, *C*_*meta*_ of P–Ph), 136.94 (s, Ar*C*), 139.01 (d, *J*_PC_ = 48.2 Hz, *C*_*ipso*_ of P–Ph), 139.49 (d, *J*_PC_ = 47.4 Hz, *C*_*ipso*_ of P–Ph), 161.26 (s, Ar*C*). ^31^P{^1^H} NMR (C_6_D_6_) *δ* 4.65 (s). ^29^Si{^1^H} NMR (C_6_D_6_) *δ* –7.20 (d, ^2^*J*_PSi_ = 3.11 Hz), –7.24 (d, ^2^*J*_PSi_ = 3.07 Hz). FTIR *ν*/cm^–1^ (Nujol): 1589 (w), 1325 (w), 1260 (s), 1197 (m), 1100 (br, s), 1095 (br, s), 1042 (m), 1023 (m), 887 (w), 856 (m), 800 (m), 726 (m), 609 (m).

### Computational details

Unrestricted and restricted geometry optimisations were performed as appropriate for full models of **1**, **2**, and **3** and the components of the exchange reactions using coordinates derived from their X-ray crystal structures. No constraints were imposed on the structures during the geometry optimisations. The calculations were performed using the Amsterdam Density Functional (ADF) suite version 2010.01.^46^,^47^ The DFT geometry optimisations employed Slater type orbital (STO) triple-ζ-plus polarisation all-electron basis sets (from the ZORA/TZP database of the ADF suite). Scalar relativistic approaches were used within the ZORA Hamiltonian for the inclusion of relativistic effects and the local density approximation (LDA) with the correlation potential due to Vosko *et al.*^48^ was used in all of the calculations. Gradient corrections were performed using the functionals of Becke^49^ and Perdew.^50^ Following geometry optimisation a single point energy (SPE) calculation was performed. MOLEKEL^51^ was used to prepare the three-dimensional plot of the electron density. Natural Bond Order (NBO) analyses were carried out with NBO 5.052 since this method is well suited to describing covalency effects in molecules.^35^,^52^ Optimisations of the L–H bonds in hydrogen terminated truncated complexes were performed using version 6.4 of the TURBOMOLE software package,^53^ employing the PBE functional,^54^ based on the generalised gradient approximation (GGA). Ahlrichs basis sets^55^ of polarised triple-ζ quality (def-TZVP for Ce, Th, U; def2-TZVP for all other atoms) were used for these partial optimisations. Total electron densities were obtained *via* single point energy (SPE) calculations, replacing the basis sets of the metal ions with the segmented all-electron relativistically contracted (SARC) basis sets,^56^ again of polarised triple-ζ quality. In these SPE calculations, scalar relativistic effects were incorporated *via* the 2nd order Douglas–Kroll–Hess Hamiltonian.^57^,^58^ Correlated electron wavefunctions of the truncated systems were obtained by employing the restricted-active-space and complete active space self-consistent-field (RASSCF/CASSCF) methodologies^59^ using version 7.6 of the MOLCAS software package.^60^,^61^ In these calculations, all-electron ANO-RCC basis sets^62^–^64^ of approximate polarised triple-ζ quality were employed, with scalar relativistic effects again incorporated *via* the 2nd order Douglas–Kroll–Hess Hamiltonian. Topological and integrated atomic properties, obtained using the quantum theory of atoms in molecules (QTAIM), were performed using version 13.11.04 of the AIMAll software package.^65^

## Supplementary Material

Supplementary information

Crystal structure data
