# Supplementary material for: Emergence of comparable covalency in isostructural cerium(iv)– and uranium(iv)–carbon multiple bonds
Source: Chem Sci. 2016 Feb 4;7(5):3286–97. doi: 10.1039/c6sc00278a (PMC6006499; doi:10.1039/c6sc00278a)
Supplement: Supplementary file 1 [file SC-007-C6SC00278A-s001.pdf]

Electronic Supplementary Material (ESI) for Chemical Science.
This journal is © The Royal Society of Chemistry 2016

- Electronic Supplementary Information -

Emergence of Comparable Covalency in Isostructural Cerium(IV)- and Uranium(IV)-Carbon Multiple Bonds

Matthew Gregson,[1†] Erli Lu,[1†] Floriana Tuna,[2] Eric J. L. McInnes,[2] Christoph Hennig,[3,4] Andreas C. Scheinost,[3,4] Jonathan McMaster,[5] William Lewis,[5] Alexander J. Blake,[5] Andrew Kerridge,*[,6] Stephen T. Liddle*[,1]

[1] School of Chemistry, The University of Manchester, Oxford Road, Manchester, M13 9PL, UK. [2] EPSRC National UK EPR Facility, School of Chemistry and Photon Science Institute, The University of Manchester, Oxford Road, Manchester, M13 9PL, UK. [3] Helmholtz-Zentrum Dresden-Rossendorf, Institute of Resource Ecology, Bautzner Landstrasse 400, D-01314 Dresden, Germany. [4] The Rossendorf Beamline, ESRF, BP 220, F-38043 Grenoble, France. [5] School of Chemistry, University of Nottingham, University Park, Nottingham, NG7 2RD, UK. [6] Department of Chemistry, Lancaster University, Lancaster, LA1 4YB, UK. *Email: steve.liddle@manchester.ac.uk; a.kerridge@lancaster.ac.uk. [†] Contributed equally.

Contents

UV/Vis/NIR Electronic Absorption Spectra……………………………………………..…………………S2

X-ray Crystallography……………………………………………………………………………………S2

Magnetic Measurements…………………………………………………………………………………S4

EPR Measurements………………………………………………………………………………………S6

DFT Calculations……………………………………………………………………………………...S6

References…………………………………………………………………………………...…….S18

UV/Vis/NIR Electronic Absorption Spectra

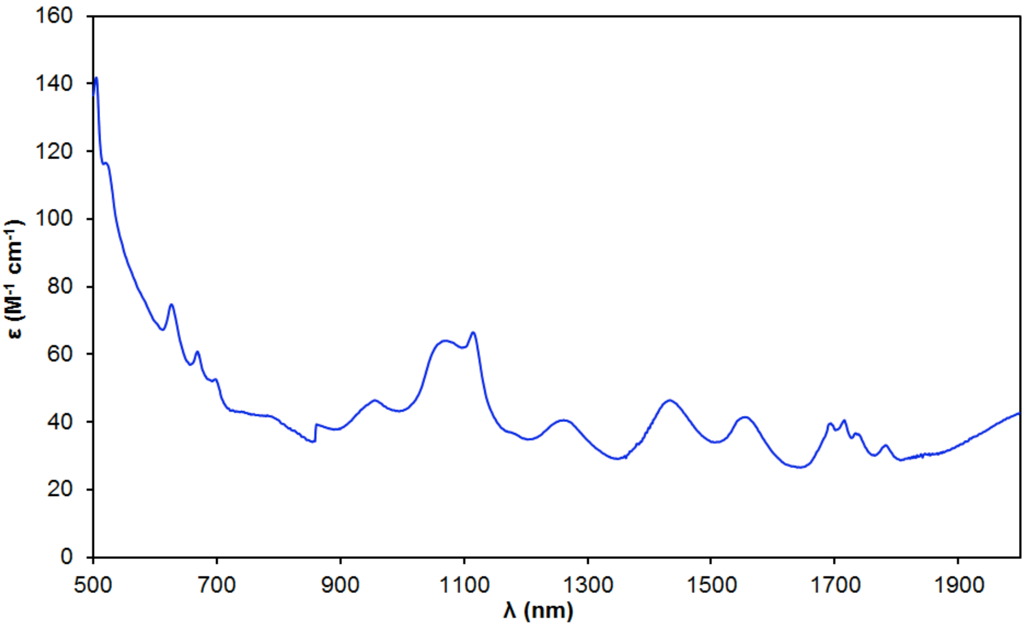

Figure S1. [U(BIPM^{TMS})(ODipp)$_2$] (2) 25mM in THF, 1mm quartz cell

The UV/Vis/NIR spectrum of [Th(BIPM^{TMS})(ODipp)$_2$] (**3**) is featureless in the range 400-2000 nm, commensurate with its colourless nature.

X-ray Crystallography

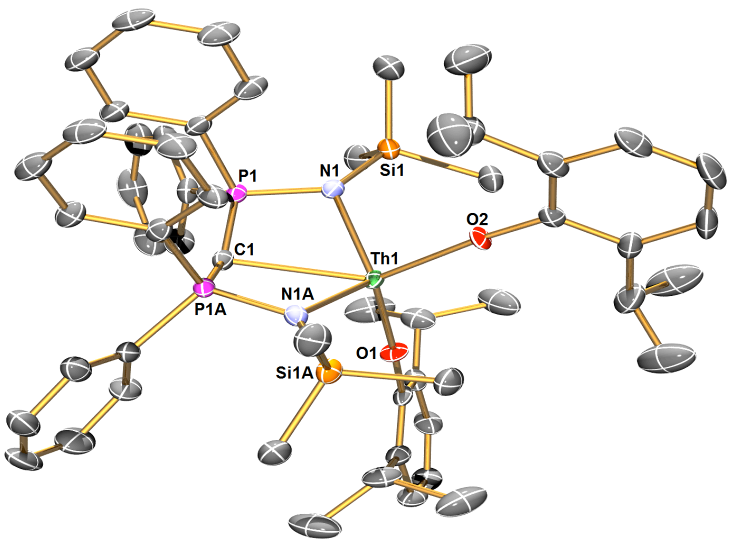

Figure S2. Molecular structure of [Th(BIPM^{TMS})(ODipp)$_2$] (3). Displacement ellipsoids set to 40%, hydrogen atoms and minor disorder components omitted for clarity.

Table S1. Experimental X-ray crystallographic details for [U(BIPM^{TMS})(ODipp)$_2$] (2) and [Th(BIPM^{TMS})(ODipp)$_2$] (3), CCDC 938905 and 938904

	**2**	**3**
Chemical formula	$C_{55}H_{72}N_2O_2P_2Si_2U{\cdot}C_4H_{10}O$	$C_{55}H_{72}N_2O_2P_2Si_2Th{\cdot}C_7H_8$
M_r	1223.41	1235.46
Crystal system, space group	Orthorhombic, *Pnma*	Orthorhombic, *Pnma*
a, b, c (Å)	18.4956 (2), 16.94471 (14), 18.98054 (16)	19.1242 (17), 16.7581 (14), 18.8909 (19)
α, β, γ (°)	90, 90, 90	90, 90, 90
V (Å^3)	5948.57 (10)	6054.3 (10)
Z	4	4
Radiation type	Cu $K\alpha$	Mo $K\alpha$
μ (mm^{-1})	8.90	2.60
Crystal size (mm)	0.45 × 0.19 × 0.17	0.13 × 0.07 × 0.03
Diffractometer	SuperNova, Single source at offset), Atlas diffractometer	Bruker *SMART APEX* CCD area detector diffractometer
Absorption correction	Gaussian *CrysAlis PRO*, Agilent Technologies, Version 1.171.36.21 Numerical absorption correction based on gaussian integration over a multifaceted crystal model	Multi-scan *SADABS* 2007/2
T_{min}, T_{max}	0.169, 0.519	0.322, 0.430
No. of measured, independent and observed [$I > 2\sigma(I)$] reflections	49347, 6226, 6196	43205, 7245, 5470
R_{int}	0.037	0.084
$(\sin \theta/\lambda)_{max}$ (Å^{-1})	0.626	0.669
$R[F^2 > 2\sigma(F^2)]$, $wR(F^2)$, S	0.022, 0.059, 1.12	0.039, 0.085, 1.10
No. of reflections	6226	7245
No. of parameters	349	360
No. of restraints	0	3
	$w = 1/[\sigma^2(F_o^2) + (0.031P)^2 + 6.7773P]$ where $P = (F_o^2 + 2F_c^2)/3$	$w = 1/[\sigma^2(F_o^2) + (0.024P)^2 + 17.6723P]$ where $P = (F_o^2 + 2F_c^2)/3$
$\Delta\rangle_{max}$, $\Delta\rangle_{min}$ (e Å^{-3})	1.36, -1.20	1.42, -3.00

Magnetic Measurements

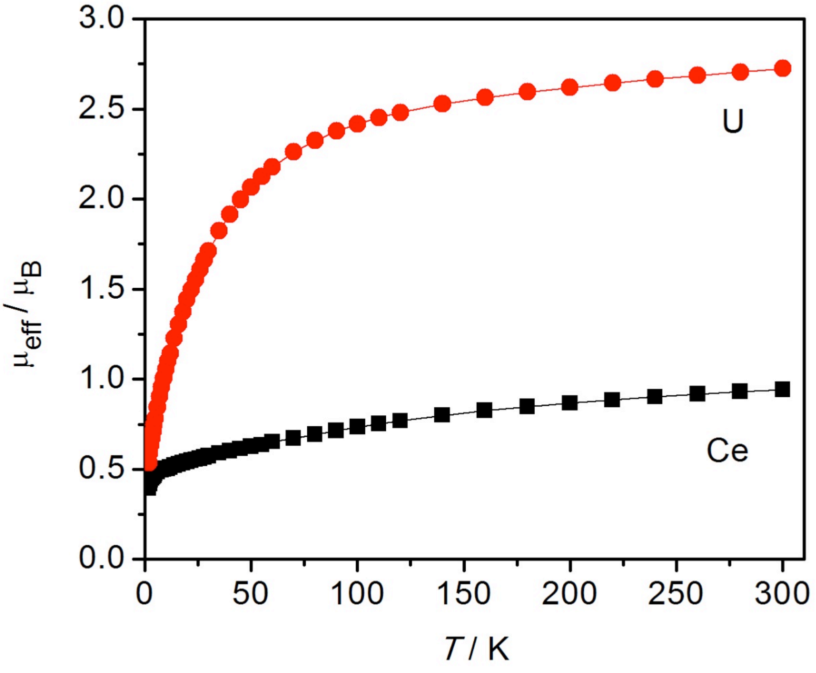

Figure S3. ***μ_{eff} vs T (K) plot for the cerium complex 1 and uranium complex 2.***

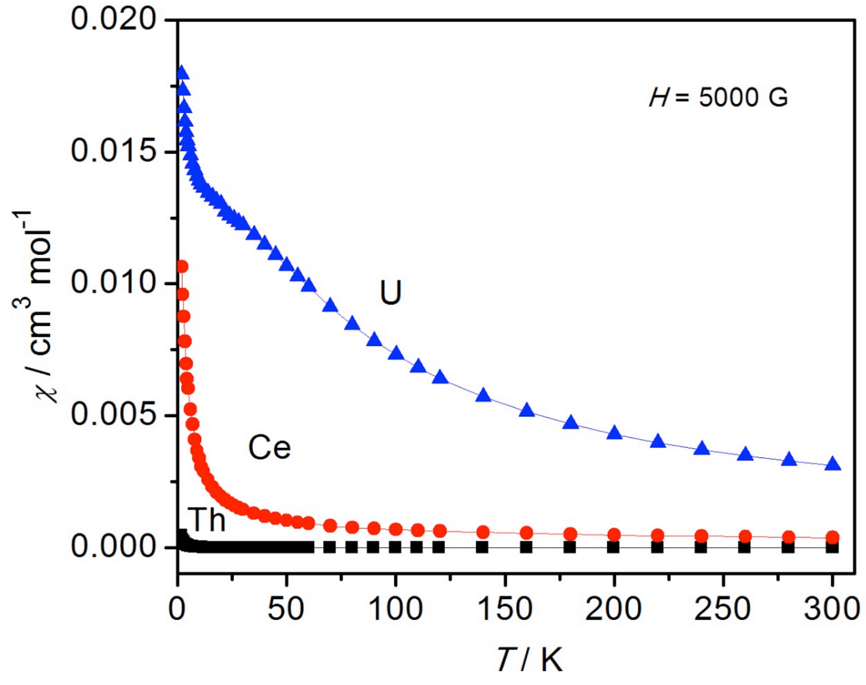

Figure S4. ***χ vs T (K) plot for the cerium complex 1, uranium complex 2, and thorium complex 3.***

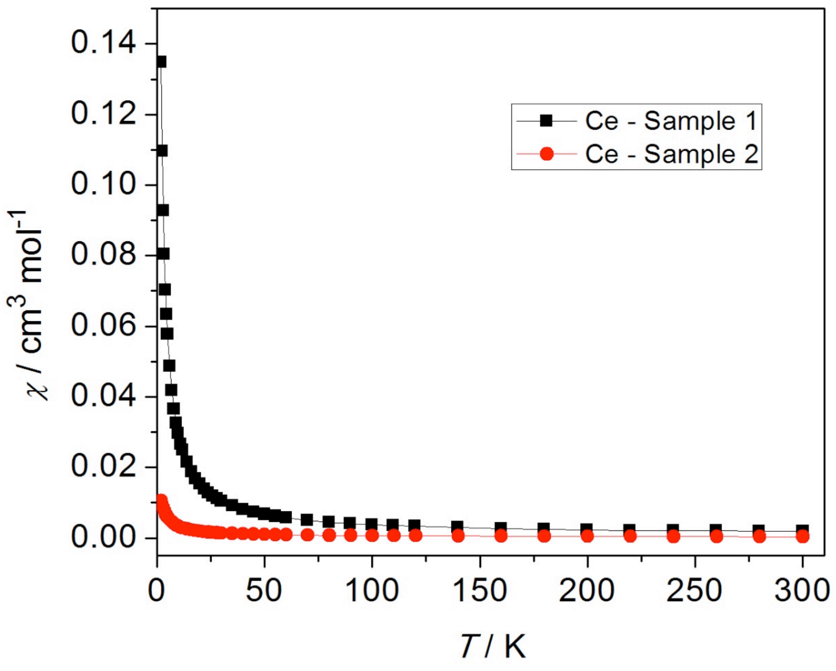

Figure S5. χ vs T (K) comparison of the variation of the cerium(III) impurity in cerium(IV) complex 1 in two independent samples.

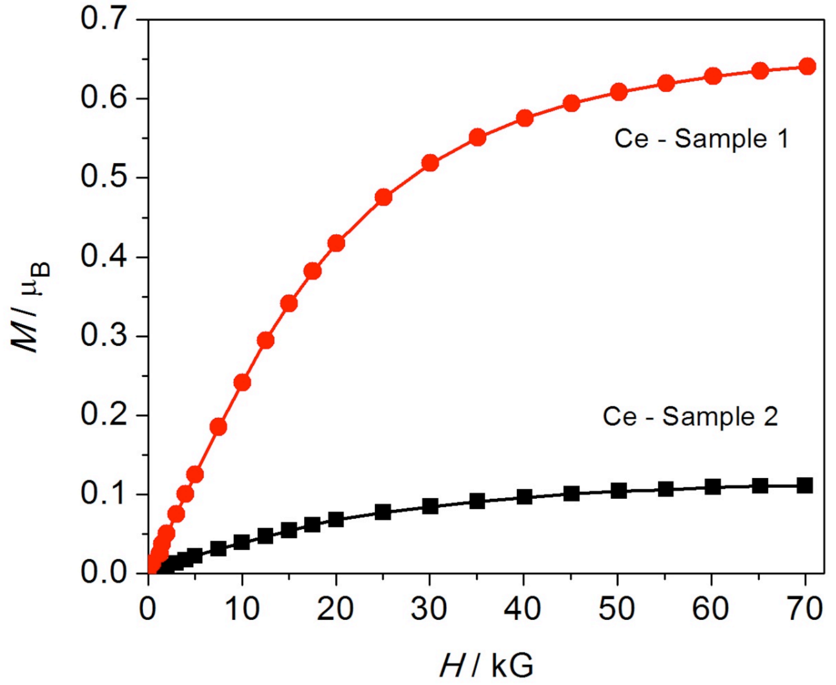

Figure S6. M vs H (kG) comparison of the variation of the cerium(III) impurity in cerium(IV) complex 1 in two independent samples.

EPR Measurements

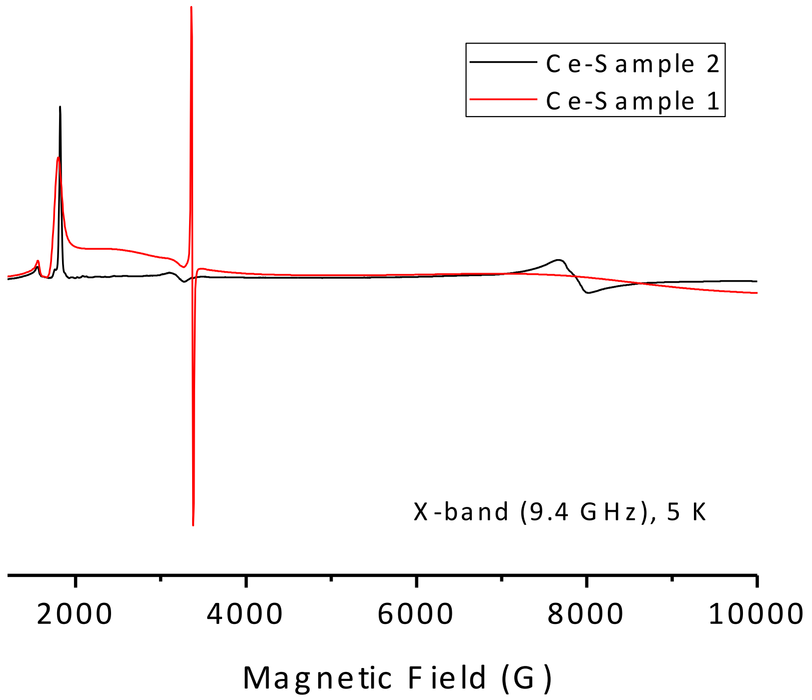

Figure S7. Comparison of the X-band EPR spectra of the two independent samples with cerium(III) impurity in cerium(IV) complex 1. For $g_{x,y}$ = 3.68; g_z = 0.85 for the cerium(III) impurity , based on EPR, a calculation of μ_{eff} with formula $4\ \mu_{eff}^2 = g_1^2 + g_2^2 + g_3^2$ gives μ_{eff}= 2.64 μ_B (as would be expected for a $^2F_{5/2}$ cerium(III) ion) whereas the value obtained from SQUID measurements is 0.9 μ_B at 298 K and 0.3 μ_B at 2 K for sample 2.

DFT Calculations

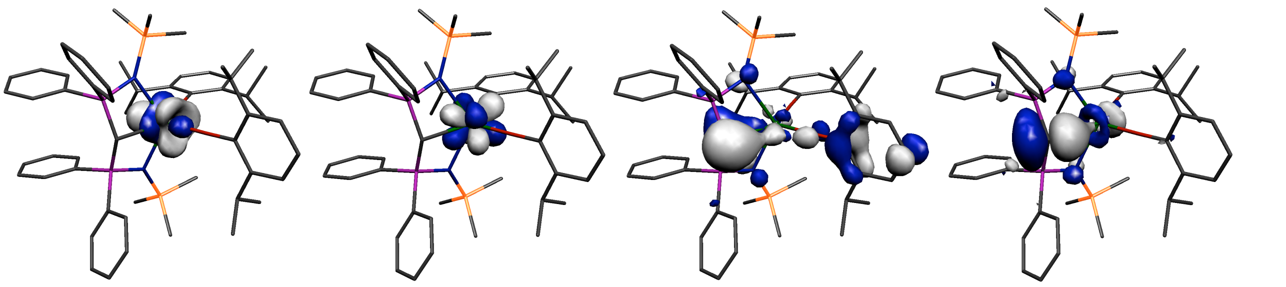

Figure S8. Selected Kohn Sham Orbitals for [U(BIPM^{TMS})(ODipp)$_2$] (2). Left to right: HOMO (292a, –2.958 eV); HOMO–1 (291a, –3.027); HOMO–4 (288a, –5.110 eV); HOMO–5 (287a, –5.382 eV).

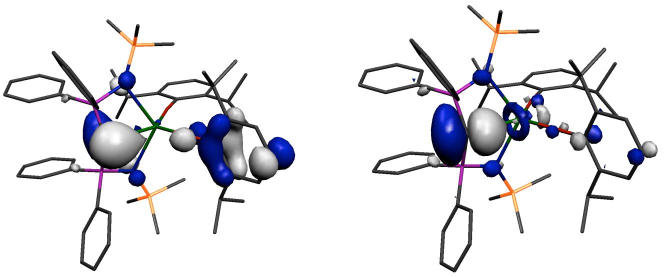

Figure S9. Selected Kohn Sham Orbitals for [Th(BIPM^{TMS})(ODipp)$_2$] (3). Left to right: HOMO–2 (288, –4.995 eV); HOMO–3 (287, –5.222 eV).

Table S2. Final Coordinates and Single Point Energy of [Ce(BIPM^{TMS})(ODipp)$_2$] (1) after Geometry Optimisation

1.H	-1.528358	-2.272888	-6.794111
2.H	-0.510760	-0.136036	-6.011719
3.C	-1.122946	-2.197505	-5.783637
4.C	-0.552835	-0.998569	-5.344916
5.H	-1.626236	-4.234661	-5.256357
6.H	-5.315166	-1.170325	-4.901383
7.C	-1.176745	-3.298822	-4.920794
8.H	-0.323043	2.318738	-4.899069
9.H	0.236701	4.004518	-4.859296
10.C	0.197694	3.067780	-4.283687
11.H	2.973566	-0.954001	-4.330569
12.C	-4.726867	-1.149849	-3.982292
13.H	-4.300452	0.966067	-4.113849
14.H	1.232020	2.732024	-4.129345
15.H	4.339585	0.163348	-4.117784
16.H	2.682831	0.795802	-4.209638
17.C	-0.032083	-0.902733	-4.051621
18.H	-4.973872	-3.262567	-3.586574
19.H	-2.517716	2.944264	-3.791238
20.C	3.295190	-0.032032	-3.824139
21.H	-2.045213	4.654733	-3.709399
22.H	0.420102	0.027145	-3.710164
23.C	-4.157553	0.048509	-3.541056
24.C	-0.659239	-3.203607	-3.626782
25.H	-0.010188	5.905769	-3.414161
26.C	-4.536238	-2.323627	-3.243566
27.H	2.006179	-3.723552	-3.294299
28.C	-1.991910	3.692241	-3.179251
29.H	3.454702	-5.679783	-2.849949
30.C	-0.070076	-2.004846	-3.184834
31.H	-0.704302	-4.067222	-2.961711
32.C	-0.530135	3.270336	-2.941827
33.C	0.384104	5.584906	-2.447318
34.H	-0.540509	2.305282	-2.417525
35.H	-2.532934	3.807723	-2.230548

36.C	2.158999	-4.056396	-2.267426
37.C	-3.406626	0.075036	-2.362773
38.H	1.183623	7.536943	-1.974547
39.C	2.980981	-5.158788	-2.016338
40.C	-3.783867	-2.299407	-2.066769
41.C	0.204759	4.256246	-2.042859
42.H	-2.968814	1.008530	-2.012640
43.H	4.417151	-2.395094	-1.957775
44.C	1.054762	6.506885	-1.642103
45.Si	3.206350	-0.179253	-1.939499
46.H	-3.642824	-3.218726	-1.496444
47.C	-3.221295	-1.093767	-1.609339
48.H	5.505817	-1.034074	-1.595830
49.H	4.722697	1.736431	-1.508323
50.H	3.036155	2.302512	-1.510490
51.C	4.500797	-1.446463	-1.407373
52.P	0.574089	-1.837190	-1.475415
53.N	1.579304	-0.534676	-1.349640
54.C	1.532913	-3.379425	-1.206557
55.C	3.697496	1.482698	-1.193963
56.C	3.199582	-5.586125	-0.702522
57.H	3.849704	-6.440537	-0.506647
58.C	0.722379	3.855825	-0.781438
59.H	-4.148193	2.771245	-0.796559
60.C	1.558314	6.097231	-0.406296
61.H	4.425849	-1.678696	-0.335310
62.H	-5.145568	1.394777	-0.276498
63.O	0.558395	2.575215	-0.370081
64.H	2.082649	6.819708	0.223032
65.H	3.702485	1.455668	-0.092797
66.C	-0.586168	-1.578810	-0.261246
67.C	1.407732	4.782044	0.050497
68.P	-2.188811	-1.039655	-0.093776
69.C	1.746450	-3.822757	0.108567
70.C	-4.526441	2.230009	0.082584
71.H	-5.086022	-1.668322	0.257649
72.C	2.581029	-4.915071	0.359540
73.H	-5.184072	2.918593	0.638138
74.Ce	0.364728	0.570339	0.399727
75.N	-2.033135	0.508171	0.456412
76.H	3.989159	3.952811	0.659187
77.H	1.251701	-3.303508	0.930372
78.H	-1.652915	3.657585	0.831644
79.C	-4.536100	-2.119855	1.083566
80.H	2.749833	-5.244605	1.385828
81.H	3.796421	5.579346	1.347098
82.C	-3.133954	-2.032150	1.127706
83.H	5.663665	0.313209	1.324749
84.Si	-3.134957	1.653335	1.228523
85.C	3.501246	4.525229	1.459886
86.C	1.970012	4.371548	1.404859
87.H	1.739476	3.306557	1.544753
88.H	5.284254	-2.186054	1.637532
89.H	3.361620	-0.602092	1.596667
90.H	-6.323940	-2.851268	2.043906
91.C	-2.100995	3.158181	1.703140
92.C	-5.235914	-2.785027	2.094357
93.H	4.630039	1.495614	2.167195
94.C	5.091478	0.501683	2.245100
95.H	-2.739209	3.892311	2.220789
96.H	3.888321	4.170368	2.426764
97.H	1.523443	6.221745	2.487951
98.C	-2.445872	-2.617809	2.202548
99.H	-1.289256	2.897997	2.400766
100.H	-1.357092	-2.559100	2.230581
101.C	4.033475	-0.595973	2.466462
102.C	4.704411	-1.979361	2.548921
103.C	1.307834	5.144953	2.560171
104.H	0.216491	5.020329	2.551695
105.O	1.250720	0.243674	2.361429
106.H	-4.715584	0.244673	2.605619
107.H	3.960160	-2.778088	2.669306
108.H	5.801198	0.530151	3.085725
109.C	-3.934716	0.992789	2.806133
110.C	-4.544083	-3.356195	3.167557
111.H	5.398624	-2.034888	3.400315
112.C	-3.147387	-3.270491	3.219741
113.H	1.691210	4.790893	3.528535
114.H	-4.401476	1.830716	3.349902
115.H	-3.194721	0.527084	3.472811
116.H	-5.091659	-3.867553	3.960966
117.C	1.817557	0.086370	3.586702
118.C	3.181701	-0.305700	3.693881
119.H	-0.744709	0.582777	3.648986
120.H	-2.603879	-3.714844	4.055055
121.H	-0.046272	2.844337	4.502808
122.C	-0.426703	0.690898	4.695462
123.C	1.045764	0.311374	4.761566
124.H	4.801591	-0.699205	5.057691
125.C	3.752211	-0.411248	4.969002
126.H	-1.706961	2.437647	5.005613
127.C	-0.648734	2.157070	5.112382
128.H	-1.181485	-1.295154	5.237683
129.H	-2.362446	0.018267	5.457086
130.C	-1.299276	-0.248263	5.548289
131.C	1.670587	0.189938	6.009320
132.H	-0.362409	2.308203	6.164177
133.C	3.015421	-0.159474	6.125730
134.H	-1.033226	-0.180950	6.613440
135.H	1.087116	0.373044	6.913729
136.H	3.482069	-0.242594	7.107387

Energy: −809.60194083 eV

Table S3. Final Coordinates and Single Point Energy of [U(BIPM^{TMS})(ODipp)$_2$] (2) after Geometry Optimisation

1.C	3.677691	0.588656	-5.023817
2.C	2.356565	-3.270892	-4.781754
3.C	2.384314	1.054692	-4.768521
4.C	-1.788067	1.654393	-4.628689
5.C	1.057512	-2.824089	-4.520709
6.C	-2.596630	-0.737598	-4.475814
7.C	4.509824	0.226461	-3.957815
8.C	3.283183	-3.365738	-3.736505
9.C	-4.720721	1.375636	-3.586112
10.C	-2.319410	0.556538	-3.688609
11.C	1.925002	1.162077	-3.453150
12.C	0.684077	-2.477898	-3.219422
13.C	-5.855155	1.804000	-2.895576
14.C	-3.545258	1.020179	-2.911285
15.C	4.052401	0.333229	-2.642198
16.C	2.911841	-3.020065	-2.435000
17.C	0.923152	4.299435	-2.458364
18.C	2.755948	0.812979	-2.378765
19.C	1.602274	-2.581119	-2.164598
20.C	-1.946278	-4.235135	-1.915407
21.C	-5.821257	1.882390	-1.502363
22.C	-3.532927	1.104721	-1.495393
23.C	-4.674386	1.539269	-0.774611
24.C	4.405414	2.576341	-0.352988
25.C	-1.277522	3.961435	-0.362661
26.C	1.924128	-4.735541	0.112766
27.C	-3.426245	-2.402936	0.036704
28.C	5.379287	3.237215	0.400828
29.C	1.596630	-0.536790	0.048728
30.C	3.498031	1.699857	0.266854
31.C	1.571824	4.644958	0.502454
32.C	1.762556	-3.424227	0.592302
33.C	-4.667690	1.634852	0.745366
34.C	2.320940	-5.761157	0.975308
35.C	-4.888872	3.079350	1.230134
36.C	-1.532435	-4.556966	1.089407
37.C	-5.697934	0.685251	1.383743
38.C	5.446167	3.043307	1.784276
39.C	3.579522	1.500732	1.654207
40.C	2.001271	-3.158575	1.949957
41.C	2.545262	-5.491130	2.329105
42.C	4.543322	2.174357	2.409211
43.C	2.384348	-4.187553	2.814340
44.C	-1.268828	4.145117	3.458159
45.C	-0.220978	3.030776	3.639795
46.C	-3.385085	-2.102051	3.857928
47.C	-1.086078	0.610800	3.796502
48.C	-1.870422	-1.837161	3.951539
49.C	1.049264	3.593201	4.303587
50.C	-0.793169	1.854378	4.417953
51.C	-1.582759	-0.474805	4.567096
52.C	-1.174413	-2.973385	4.722984
53.C	-1.058630	1.999262	5.786757
54.C	-1.828117	-0.271273	5.932027
55.C	-1.581210	0.954798	6.547452
56.H	4.034600	0.500314	-6.051410
57.H	2.649291	-3.538646	-5.798571
58.H	1.730238	1.335964	-5.595284
59.H	-2.529894	1.911120	-5.399632
60.H	0.331234	-2.746787	-5.331325
61.H	-3.373142	-0.580004	-5.239145
62.H	-0.876159	1.315748	-5.142783
63.H	-1.686551	-1.080027	-4.990793
64.H	-4.747711	1.315367	-4.676394
65.H	5.516840	-0.146284	-4.151836
66.H	-1.553813	2.574577	-4.076778
67.H	4.300660	-3.704485	-3.937398
68.H	-2.943495	-1.541810	-3.812914
69.H	-6.759917	2.075094	-3.439623
70.H	0.919476	1.527261	-3.251132
71.H	0.261632	3.868396	-3.223357
72.H	-1.533995	0.338046	-2.951014
73.H	-0.327377	-2.133012	-3.011863
74.H	1.962347	4.107494	-2.764075
75.H	-2.246028	-3.585692	-2.750387
76.H	0.770001	5.391210	-2.464882
77.H	-1.010341	-4.738482	-2.199156
78.H	4.708434	0.050700	-1.817557
79.H	-2.721796	-5.010950	-1.803972
80.H	3.640313	-3.097798	-1.626595
81.H	4.356512	2.742886	-1.429298
82.H	-6.709258	2.218965	-0.963246
83.H	1.742010	-4.959636	-0.938517
84.H	-1.944612	3.474856	-1.088205
85.H	-3.690956	-1.698516	-0.764676
86.H	-1.476205	5.044460	-0.396284
87.H	6.082889	3.907533	-0.095279
88.H	-4.237633	-3.143843	0.115373
89.H	2.632262	4.703426	0.216184
90.H	2.448938	-6.772990	0.587776
91.H	1.177976	5.673867	0.548325
92.H	-1.562836	3.617913	0.644254
93.H	-5.883834	3.447038	0.936811
94.H	-4.140675	3.762628	0.805786
95.H	-0.731249	-5.273322	0.854139
96.H	-6.723129	0.961823	1.094891
97.H	-5.530350	-0.354573	1.071201
98.H	-3.411375	-1.846475	0.986996
99.H	-3.671634	1.319931	1.087337
100.H	-2.463371	-5.128888	1.235643
101.H	1.526489	4.220101	1.515405

102.H	6.200558	3.566803	2.373904
103.H	-1.275437	-4.077717	2.045025
104.H	-4.822907	3.129655	2.326886
105.H	2.882223	0.812578	2.133087
106.H	-5.634055	0.732411	2.481063
107.H	1.885534	-2.139060	2.319263
108.H	2.845222	-6.294319	3.004048
109.H	0.058249	2.658849	2.643693
110.H	-0.866170	4.964740	2.844571
111.H	-2.179483	3.766600	2.973857
112.H	-1.462301	-1.827190	2.930754
113.H	4.591734	2.021415	3.488322
114.H	-3.584496	-3.062016	3.359642
115.H	-3.898989	-1.308597	3.297877
116.H	2.557756	-3.972840	3.869822
117.H	1.465410	4.420501	3.710270
118.H	-1.561296	4.563724	4.432900
119.H	-1.364674	-3.944173	4.241350
120.H	1.823725	2.821015	4.405384
121.H	-3.833719	-2.142044	4.862289
122.H	-0.087626	-2.820491	4.767170
123.H	0.832742	3.986352	5.307831
124.H	-1.546996	-3.040756	5.755405
125.H	-0.850282	2.956916	6.267622
126.H	-2.223624	-1.096569	6.527256
127.H	-1.786173	1.091976	7.609165
128.N	0.781421	1.892072	-0.581391
129.N	-0.546382	-2.029181	-0.331898
130.O	-2.402045	0.763492	-0.818407
131.O	-0.890823	0.458410	2.452866
132.P	2.120450	0.918052	-0.662995
133.P	1.105045	-2.080652	-0.472970
134.Si	0.545214	3.642677	-0.725331
135.Si	-1.792848	-3.288393	-0.284962
136.U	-0.668587	0.245994	0.314732

Energy: −811.58109884 eV

Table S4. Final Coordinates and Single Point Energy of [Th(BIPM^{TMS})(ODipp)$_2$] (3) after Geometry Optimisation

1.C	4.268709	0.748405	-5.333586
2.C	2.986813	0.204847	-5.421023
3.C	-0.132297	0.337001	-5.335523
4.C	0.813303	-1.972220	-4.919537
5.C	0.769379	-0.515119	-4.423118
6.C	2.165346	0.080797	-4.292586
7.C	4.744322	1.178394	-4.094207
8.C	-3.938618	0.007037	-3.730472
9.C	-5.223302	-0.437627	-3.404752
10.C	-2.347607	-3.867077	-3.003593
11.C	2.665386	0.521879	-3.040846
12.C	3.966655	1.078041	-2.932879
13.C	-3.618786	-4.345050	-2.672026
14.C	-1.909946	3.645463	-2.916195
15.C	-3.053022	0.392799	-2.720461
16.C	-5.616258	-0.503652	-2.062555
17.C	-1.578302	-3.198499	-2.046948
18.C	4.832700	3.069169	-1.617739
19.C	4.524729	1.560611	-1.599911
20.C	-4.121981	-4.143530	-1.381064
21.C	0.874849	3.724445	-1.651677
22.C	5.768291	0.759443	-1.172200
23.C	-3.442424	0.343570	-1.373957
24.C	1.484541	-4.628955	-1.353108
25.C	-4.731253	-0.120912	-1.051914
26.C	-2.068153	-3.004915	-0.747099
27.C	-3.355257	-3.473872	-0.424765
28.C	3.381644	-2.387667	-0.497441
29.C	-1.538878	4.721631	-0.085962
30.C	-4.336524	2.603579	0.648841
31.C	-1.427503	-0.451747	0.714690
32.C	-3.267353	1.810643	1.100801
33.C	-5.040127	3.419308	1.539111
34.C	2.118206	-4.196829	1.600671
35.C	2.316063	4.827945	1.780199
36.C	-1.442308	-4.477757	1.975416
37.C	-1.265666	-3.083740	2.017678
38.C	-2.910431	1.855761	2.457244
39.C	1.388434	3.876223	2.560844
40.C	-4.673133	3.464178	2.888533
41.C	4.722042	-1.063352	2.899899
42.C	-1.513025	-5.219677	3.157569
43.C	2.355353	1.535222	3.016507
44.C	-3.604340	2.683489	3.344799
45.C	-1.152450	-2.448073	3.263552
46.C	0.383094	4.679567	3.404580
47.C	2.194006	2.893499	3.399582
48.C	3.305930	-0.816110	3.456157
49.C	3.111942	0.647845	3.827858
50.C	-1.391114	-4.579305	4.395652
51.C	-1.206718	-3.192479	4.445559
52.C	2.824902	3.340639	4.568993
53.C	3.020947	-1.762302	4.635877
54.C	3.719735	1.151291	4.986581
55.C	3.588741	2.486834	5.362401
56.H	2.613462	-0.132111	-6.390624
57.H	4.891925	0.836181	-6.223263
58.H	0.262682	0.370616	-6.361917
59.H	1.248144	-2.033908	-5.928308
60.H	-1.147434	-0.084578	-5.381271
61.H	-0.201926	1.372386	-4.975328
62.H	-0.200474	-2.397202	-4.965754
63.H	-3.626299	0.059044	-4.774574
64.H	1.424171	-2.601614	-4.258039

65.H	5.747268	1.604780	-4.024721
66.H	-5.915773	-0.735608	-4.194083
67.H	-1.949728	-4.020424	-4.007974
68.H	-4.217772	-4.870945	-3.417687
69.H	-1.519080	3.021056	-3.732552
70.H	0.323914	-0.516826	-3.418910
71.H	-1.819429	4.698044	-3.231219
72.H	-2.055908	0.749385	-2.974335
73.H	-2.981425	3.422351	-2.805844
74.H	5.616591	3.300722	-2.354196
75.H	-0.583613	-2.835746	-2.301802
76.H	1.446341	-4.205572	-2.367199
77.H	1.290416	3.038250	-2.404347
78.H	3.942113	3.656514	-1.880020
79.H	-6.616006	-0.854478	-1.801797
80.H	6.590502	0.898807	-1.890001
81.H	1.001740	4.750980	-2.031598
82.H	-5.115176	-4.510816	-1.117835
83.H	5.552427	-0.316170	-1.115072
84.H	2.307634	-5.362050	-1.330648
85.H	0.546312	-5.177110	-1.182139
86.H	3.311044	-1.887829	-1.474811
87.H	5.190787	3.401990	-0.631949
88.H	3.747861	1.392590	-0.838763
89.H	1.493199	3.655302	-0.740800
90.H	6.123463	1.095902	-0.186649
91.H	4.220675	-3.100169	-0.548328
92.H	-4.626684	2.581240	-0.401980
93.H	-1.236486	5.714980	-0.456561
94.H	-5.048729	-0.170830	-0.009266
95.H	-2.631153	4.728276	0.041739
96.H	3.663268	-1.634569	0.257715
97.H	-3.753748	-3.327404	0.580360
98.H	-5.873957	4.022028	1.175592
99.H	-1.092287	4.581650	0.908996
100.H	-1.532798	-4.987835	1.015967
101.H	1.734010	5.505034	1.137407
102.H	3.023623	4.273992	1.147557
103.H	3.061694	-4.762559	1.526937
104.H	1.319924	-4.906431	1.862692
105.H	0.813359	3.288012	1.830914
106.H	4.936070	-0.417836	2.036725
107.H	2.907161	5.443980	2.474765
108.H	2.214274	-3.486851	2.434750
109.H	4.844033	-2.111072	2.587161
110.H	-0.212105	5.347995	2.765668
111.H	-1.661722	-6.299519	3.109274
112.H	2.582820	-1.050392	2.661204
113.H	-2.089559	1.229108	2.807734
114.H	-5.219819	4.104199	3.582972
115.H	-1.030959	-1.364702	3.293733
116.H	5.479861	-0.848276	3.668521
117.H	-0.307127	4.017326	3.944265
118.H	0.895400	5.309465	4.146476
119.H	-3.315368	2.715292	4.396457
120.H	3.129048	-2.811893	4.326294
121.H	2.714687	4.385727	4.863904
122.H	-1.442770	-5.158935	5.318933
123.H	2.003033	-1.622934	5.024648
124.H	-1.112376	-2.688767	5.408942
125.H	3.724292	-1.593269	5.464188
126.H	4.311766	0.478211	5.609289
127.H	4.072080	2.856917	6.266585
128.N	-1.037508	1.755368	-0.675556
129.N	0.535878	-2.080978	0.046876
130.O	1.885553	0.410079	-1.930167
131.O	1.778098	1.080224	1.862403
132.Si	-0.954089	3.403399	-1.302588
133.Si	1.799557	-3.310673	-0.034366
134.P	-2.252564	0.808940	-0.057599
135.P	-1.064419	-2.089607	0.486032
136.Th	0.823421	0.337450	0.001908

Energy: −811.37565267 eV

Table S5. Final Coordinates and Single Point Energy of [Ce(Cl)$_4$(THF)$_3$] after Geometry Optimisation

1.C	2.802722	-1.060864	-3.709110
2.C	1.442097	-0.472704	-3.359701
3.C	2.928507	-2.211759	-2.697252
4.C	2.315737	-1.605284	-1.436584
5.C	-1.317705	-1.952954	-0.731861
6.C	-1.744998	-3.195612	0.045699
7.C	-0.623469	-3.334779	1.088265
8.C	-0.354075	-1.881371	1.461318
9.C	-2.489070	1.068410	1.736469
10.C	-3.045525	2.081765	2.728087
11.C	-0.700137	1.821330	3.143182
12.C	-2.027472	2.002398	3.879565
13.O	1.314463	-0.636066	-1.902729
14.O	-0.580413	-1.118981	0.228486
15.O	-1.031424	1.136490	1.880255
16.Cl	-1.573121	1.289462	-1.950749
17.Cl	2.200607	2.395095	-1.815876
18.Cl	-0.032110	3.714772	0.414295
19.Cl	2.269059	0.574912	1.440692
20.Ce	0.441496	1.273707	-0.292907
21.H	2.853859	-1.396011	-4.753610
22.H	3.589507	-0.310952	-3.541380
23.H	0.613949	-1.026121	-3.831651
24.H	1.348232	0.596702	-3.579644
25.H	2.349224	-3.085031	-3.033656
26.H	3.965181	-2.531415	-2.529381
27.H	-0.631596	-2.190038	-1.558416

28.H	1.795587	-2.333856	-0.799772
29.H	-2.146278	-1.351409	-1.125498
30.H	3.055908	-1.064379	-0.830425
31.H	-1.845489	-4.073211	-0.605934
32.H	-2.711276	-3.028091	0.544607
33.H	0.270143	-3.790143	0.634840
34.H	-2.735854	1.279308	0.688692
35.H	-2.805661	0.041154	1.984238
36.H	-0.909772	-3.938928	1.958871
37.H	0.669297	-1.668862	1.793234
38.H	-1.059187	-1.516982	2.224426
39.H	-3.046602	3.085643	2.281181
40.H	-4.069371	1.833538	3.039036
41.H	-0.237508	2.783537	2.879986
42.H	0.029165	1.193641	3.672916
43.H	-2.021483	2.904900	4.505298
44.H	-2.242256	1.138651	4.527294

Energy: −235.31747857 eV

Table S6. Final Coordinates and Single Point Energy of [U(Cl)$_4$(THF)$_3$] after Geometry Optimisation

1.C	2.750522	-1.013710	-3.710094
2.C	1.368558	-0.500425	-3.333623
3.C	2.966338	-2.147081	-2.694117
4.C	2.359682	-1.560952	-1.420990
5.C	-1.306178	-1.952851	-0.721073
6.C	-1.736261	-3.190165	0.061878
7.C	-0.615419	-3.329248	1.105068
8.C	-0.338158	-1.877026	1.473303
9.C	-2.518072	1.079846	1.691499
10.C	-3.047092	2.071922	2.719424
11.C	-0.699792	1.777459	3.099122
12.C	-2.016795	1.946079	3.855521
13.O	1.289872	-0.653759	-1.869361
14.O	-0.564958	-1.118070	0.237490
15.O	-1.054833	1.131632	1.817504
16.Cl	-1.558506	1.338125	-1.952355
17.Cl	2.209288	2.336264	-1.838994
18.Cl	0.032655	3.703112	0.435555
19.Cl	2.262454	0.626618	1.471373
20.U	0.442403	1.253480	-0.288736
21.H	2.797822	-1.352976	-4.753676
22.H	3.494304	-0.217504	-3.563524
23.H	0.560385	-1.106551	-3.775376
24.H	1.208143	0.558444	-3.560204
25.H	2.424593	-3.053138	-3.005433
26.H	4.023353	-2.409764	-2.554514
27.H	-0.620355	-2.194075	-1.545386
28.H	1.899993	-2.308996	-0.760206
29.H	-2.132124	-1.347792	-1.115313
30.H	3.085986	-0.969835	-0.845512
31.H	-1.837356	-4.070353	-0.586833
32.H	-2.703278	-3.020419	0.559027
33.H	0.276242	-3.791234	0.653670
34.H	-2.774071	1.324288	0.652734
35.H	-2.840436	0.051041	1.916013
36.H	-0.904626	-3.928866	1.977935
37.H	0.688026	-1.668087	1.800324
38.H	-1.038697	-1.505040	2.236513
39.H	-3.037273	3.088725	2.301500
40.H	-4.069722	1.831185	3.036253
41.H	-0.231863	2.739314	2.856225
42.H	0.030505	1.125148	3.597732
43.H	-1.994234	2.827112	4.508675
44.H	-2.234260	1.064022	4.477883

Energy: −237.19695106 eV

Table S7. Final Coordinates and Single Point Energy of [Th(Cl)$_4$(THF)$_3$] after Geometry Optimisation

1.C	2.784886	-1.076051	-3.714161
2.C	1.423984	-0.501969	-3.348456
3.C	2.933143	-2.227662	-2.706026
4.C	2.331948	-1.632473	-1.433754
5.C	-1.303318	-1.981951	-0.727319
6.C	-1.738440	-3.217396	0.056365
7.C	-0.621775	-3.356143	1.103817
8.C	-0.348832	-1.903114	1.473453
9.C	-2.523489	1.062379	1.728574
10.C	-3.066147	2.078272	2.723914
11.C	-0.722297	1.788703	3.142790
12.C	-2.049834	1.978492	3.875807
13.O	1.330920	-0.649268	-1.884469
14.O	-0.565315	-1.144192	0.233387
15.O	-1.061729	1.133390	1.861167
16.Cl	-1.635553	1.237942	-1.959573
17.Cl	2.243763	2.410058	-1.876105
18.Cl	-0.017527	3.771795	0.463323
19.Cl	2.284974	0.496171	1.494132
20.Th	0.454378	1.286486	-0.294579
21.H	2.827349	-1.409074	-4.759487
22.H	3.564053	-0.317160	-3.553549
23.H	0.593677	-1.071911	-3.795374
24.H	1.313432	0.563773	-3.576968
25.H	2.357802	-3.105479	-3.037382
26.H	3.974664	-2.538941	-2.552620
27.H	-0.613958	-2.227515	-1.548072
28.H	1.811513	-2.365997	-0.803449
29.H	-2.125798	-1.377656	-1.127632
30.H	3.075910	-1.100596	-0.825092
31.H	-1.839918	-4.098268	-0.590935
32.H	-2.706182	-3.043113	0.550036
33.H	0.272369	-3.816042	0.656348
34.H	-2.777480	1.270342	0.681733

35.H	-2.837541	0.036342	1.983469
36.H	-0.913777	-3.956108	1.975370
37.H	0.673221	-1.692427	1.809350
38.H	-1.058462	-1.532773	2.229091
39.H	-3.053196	3.083533	2.281774
40.H	-4.092332	1.841974	3.034265
41.H	-0.243846	2.746185	2.895731
42.H	-0.006889	1.137175	3.660539
43.H	-2.034342	2.875480	4.507910
44.H	-2.276629	1.113095	4.515966

Energy: −237.57835488 eV

Table S8. Final Coordinates and Single Point Energy of [Ce(Cl)$_4$(HMPA)$_2$] after Geometry Optimisation

1.C	4.611739	0.986250	-3.766884
2.C	2.206435	1.510973	-3.712947
3.C	4.252482	-1.541522	-1.594208
4.C	5.386151	2.810448	-0.909345
5.C	-4.890478	0.383735	-0.470024
6.C	-3.167553	-3.397372	0.008344
7.C	3.167553	3.397372	-0.008344
8.C	4.890478	-0.383735	0.470024
9.C	-5.386151	-2.810448	0.909345
10.C	-4.252482	1.541522	1.594208
11.C	-4.611739	-0.986250	3.766884
12.C	-2.206435	-1.510973	3.712947
13.N	3.377824	1.010670	-2.976892
14.N	4.419767	-0.235772	-0.922510
15.N	4.046052	2.342521	-0.541350
16.N	-4.046052	-2.342521	0.541350
17.N	-4.419767	0.235772	0.922510
18.N	-3.377824	-1.010670	2.976892
19.O	1.928541	0.924649	-0.842367
20.O	-1.928541	-0.924649	0.842367
21.P	3.384543	1.008455	-1.306566
22.P	-3.384543	-1.008455	1.306566
23.Cl	-1.361536	1.214214	-1.892789
24.Cl	0.296625	-2.094849	-1.549444
25.Cl	-0.296625	2.094849	1.549444
26.Cl	1.361536	-1.214214	1.892789
27.Ce	0.000000	0.000000	0.000000
28.H	4.477194	0.321074	-4.634301
29.H	2.064857	0.896065	-4.614709
30.H	4.879215	1.989044	-4.142767
31.H	5.443168	0.601277	-3.167112
32.H	2.342967	2.560749	-4.024040
33.H	1.303726	1.432643	-3.099238
34.H	3.860523	-1.401691	-2.605211
35.H	5.356662	3.579480	-1.701544
36.H	3.553884	-2.186728	-1.039118
37.H	5.232995	-2.036975	-1.652684
38.H	-4.190234	0.995754	-1.059497
39.H	6.003273	1.972809	-1.253120
40.H	-2.243573	-2.962895	-0.385338
41.H	5.872132	3.250228	-0.025390
42.H	-4.982323	-0.598644	-0.941773
43.H	-5.876931	0.870785	-0.458119
44.H	-3.694841	-3.904566	-0.813573
45.H	2.912799	4.150317	-0.774098
46.H	-5.872132	-3.250228	0.025390
47.H	5.876931	-0.870785	0.458119
48.H	3.694841	3.904566	0.813573
49.H	-6.003273	-1.972809	1.253120
50.H	-2.912799	-4.150317	0.774098
51.H	4.982323	0.598644	0.941773
52.H	2.243573	2.962895	0.385338
53.H	-5.232995	2.036975	1.652684
54.H	4.190234	-0.995754	1.059497
55.H	-3.553884	2.186728	1.039118
56.H	-5.356662	-3.579480	1.701544
57.H	-3.860523	1.401691	2.605211
58.H	-4.879215	-1.989044	4.142767
59.H	-5.443168	-0.601277	3.167112
60.H	-2.342967	-2.560749	4.024040
61.H	-1.303726	-1.432643	3.099238
62.H	-4.477194	-0.321074	4.634301
63.H	-2.064857	-0.896065	4.614709

Energy: −335.13257004 eV

Table S9. Final Coordinates and Single Point Energy of [U(Cl)$_4$(HMPA)$_2$] after Geometry Optimisation

1.C	4.566372	0.951587	-3.754532
2.C	2.159497	1.470313	-3.702570
3.C	4.190146	-1.556833	-1.545613
4.C	5.359623	2.799812	-0.900351
5.C	-4.851335	0.376096	-0.500890
6.C	-3.139962	-3.403540	0.003837
7.C	3.139962	3.403540	-0.003837
8.C	4.851335	-0.376096	0.500890
9.C	-5.359623	-2.799812	0.900351
10.C	-4.190146	1.556833	1.545613
11.C	-4.566372	-0.951587	3.754532
12.C	-2.159497	-1.470313	3.702570
13.N	3.335055	0.988034	-2.960037
14.N	4.368433	-0.244053	-0.889830
15.N	4.015281	2.342221	-0.532602
16.N	-4.015281	-2.342221	0.532602
17.N	-4.368433	0.244053	0.889830
18.N	-3.335055	-0.988034	2.960037
19.O	1.891207	0.944958	-0.827826
20.O	-1.891207	-0.944958	0.827826
21.P	3.353216	1.006184	-1.291449

22.P	-3.353216	-1.006184	1.291449
23.Cl	-1.377312	1.233350	-1.876671
24.Cl	0.306381	-2.075804	-1.582763
25.Cl	-0.306381	2.075804	1.582763
26.Cl	1.377312	-1.233350	1.876671
27.U	0.000000	0.000000	0.000000
28.H	4.435822	0.259037	-4.601046
29.H	2.028434	0.846044	-4.599411
30.H	4.820763	1.945924	-4.160627
31.H	5.404011	0.592297	-3.147208
32.H	2.285675	2.519000	-4.021899
33.H	1.254275	1.387107	-3.093353
34.H	3.774475	-1.428279	-2.548316
35.H	5.340278	3.552225	-1.708905
36.H	3.503842	-2.194485	-0.968887
37.H	5.169872	-2.050975	-1.620574
38.H	-4.150220	0.974195	-1.102499
39.H	5.977204	1.953165	-1.221502
40.H	-2.224035	-2.973851	-0.413401
41.H	5.839542	3.255215	-0.021304
42.H	-4.952822	-0.611971	-0.958261
43.H	-5.834721	0.868881	-0.486347
44.H	-3.680400	-3.924265	-0.800585
45.H	2.870457	4.143761	-0.776777
46.H	-5.839542	-3.255215	0.021304
47.H	5.834721	-0.868881	0.486347
48.H	3.680400	3.924265	0.800585
49.H	-5.977204	-1.953165	1.221502
50.H	-2.870457	-4.143761	0.776777
51.H	4.952822	0.611971	0.958261
52.H	2.224035	2.973851	0.413401
53.H	-5.169872	2.050975	1.620574
54.H	4.150220	-0.974195	1.102499
55.H	-3.503842	2.194485	0.968887
56.H	-5.340278	-3.552225	1.708905
57.H	-3.774475	1.428279	2.548316
58.H	-4.820763	-1.945924	4.160627
59.H	-5.404011	-0.592297	3.147208
60.H	-2.285675	-2.519000	4.021899
61.H	-1.254275	-1.387107	3.093353
62.H	-4.435822	-0.259037	4.601046
63.H	-2.028434	-0.846044	4.599411

Energy: −337.03516528 eV

Table S10. Final Coordinates and Single Point Energy of [Th(Cl)$_4$(HMPA)$_2$] after Geometry Optimisation

1.C	4.638522	0.988332	-3.778069
2.C	2.236267	1.528055	-3.732721
3.C	4.246770	-1.536199	-1.601126
4.C	5.423420	2.805054	-0.899354
5.C	-4.907050	0.379268	-0.459088
6.C	-3.203346	-3.412379	0.013382
7.C	3.203346	3.412379	-0.013382
8.C	4.907050	-0.379268	0.459088
9.C	-5.423420	-2.805054	0.899354
10.C	-4.246770	1.536199	1.601126
11.C	-4.638522	-0.988332	3.778069
12.C	-2.236267	-1.528055	3.732721
13.N	3.404435	1.029026	-2.989412
14.N	4.426539	-0.230817	-0.930947
15.N	4.074233	2.350416	-0.545778
16.N	-4.074233	-2.350416	0.545778
17.N	-4.426539	0.230817	0.930947
18.N	-3.404435	-1.029026	2.989412
19.O	1.948215	0.951554	-0.865389
20.O	-1.948215	-0.951554	0.865389
21.P	3.411300	1.024730	-1.321083
22.P	-3.411300	-1.024730	1.321083
23.Cl	-1.406235	1.231734	-1.939619
24.Cl	0.310769	-2.154005	-1.578567
25.Cl	-0.310769	2.154005	1.578567
26.Cl	1.406235	-1.231734	1.939619
27.Th	0.000000	0.000000	0.000000
28.H	4.500800	0.314053	-4.637558
29.H	2.092392	0.903525	-4.627049
30.H	4.911407	1.985255	-4.164450
31.H	5.467231	0.605429	-3.173456
32.H	2.380159	2.573089	-4.054627
33.H	1.331265	1.462234	-3.121671
34.H	3.834562	-1.396374	-2.604202
35.H	5.409625	3.575821	-1.689551
36.H	3.557949	-2.180324	-1.033204
37.H	5.225581	-2.032466	-1.678188
38.H	-4.210423	0.992516	-1.050935
39.H	6.034727	1.961839	-1.238754
40.H	-2.271320	-2.988534	-0.371784
41.H	5.905441	3.236850	-0.009465
42.H	-4.999064	-0.602375	-0.931333
43.H	-5.893791	0.864123	-0.440874
44.H	-3.729140	-3.907013	-0.816235
45.H	2.964646	4.173569	-0.775486
46.H	-5.905441	-3.236850	0.009465
47.H	5.893791	-0.864123	0.440874
48.H	3.729140	3.907013	0.816235
49.H	-6.034727	-1.961839	1.238754
50.H	-2.964646	-4.173569	0.775486
51.H	4.999064	0.602375	0.931333
52.H	2.271320	2.988534	0.371784
53.H	-5.225581	2.032466	1.678188
54.H	4.210423	-0.992516	1.050935
55.H	-3.557949	2.180324	1.033204
56.H	-5.409625	-3.575821	1.689551
57.H	-3.834562	1.396374	2.604202

58.H	-4.911407	-1.985255	4.164450
59.H	-5.467231	-0.605429	3.173456
60.H	-2.380159	-2.573089	4.054627
61.H	-1.331265	-1.462234	3.121671
62.H	-4.500800	-0.314053	4.637558
63.H	-2.092392	-0.903525	4.627049

Energy: −337.36202886 eV

Table S11. Coordinates of truncated Ce complex used in MCSCF calculations

1 Ce	0.159856	-0.211671	0.000000
2 C	-2.256854	-0.557266	0.000000
3 C	2.528546	2.438551	0.000000
4 C	1.653058	-3.458001	0.000000
5 N	-0.901654	0.325364	2.082946
6 N	-0.901654	0.325364	-2.082946
7 O	1.067337	-2.190363	0.000000
8 O	1.575875	1.414529	0.000000
9 P	-2.443163	0.001107	1.591592
10 P	-2.443163	0.001107	-1.591592
11 Si	-0.144052	0.578592	3.658842
12 Si	-0.144052	0.578592	-3.658842
13 H	1.308961	0.676588	3.417162
14 H	1.308961	0.676588	-3.417162
15 H	-0.582467	1.800847	4.328080
16 H	-0.582467	1.800847	-4.328080
17 H	-0.343253	-0.542222	4.603514
18 H	-0.343253	-0.542222	-4.603514
19 H	-2.982374	-0.941344	2.405973
20 H	-2.982374	-0.941344	-2.405973
21 H	-3.233579	1.074196	1.766660
22 H	-3.233579	1.074196	-1.766660
23 H	3.555510	2.028274	0.000000
24 H	2.421096	3.080242	0.894296
25 H	2.421096	3.080242	-0.894296
26 H	2.757460	-3.389056	0.000000
27 H	1.351947	-4.037464	0.892979
28 H	1.351947	-4.037464	-0.892979

Table S12. Coordinates of truncated U complex used in MCSCF calculations

1 U	-0.198849	0.153671	0.000000
2 C	-0.562491	-2.230120	0.000000
3 C	2.405856	2.547879	0.000000
4 C	-3.496045	1.496216	0.000000
5 N	0.327905	-0.868011	2.072516
6 N	0.327905	-0.868011	-2.072516
7 O	1.365851	1.605689	0.000000
8 O	-2.195584	0.977788	0.000000
9 P	0.025798	-2.422357	1.581484
10 P	0.025798	-2.422357	-1.581484
11 Si	0.577778	-0.125930	3.662487
12 Si	0.577778	-0.125930	-3.662487
13 H	0.687438	1.321248	3.428931
14 H	0.687438	1.321248	-3.428931
15 H	-0.533034	-0.354826	4.597689
16 H	-0.533034	-0.354826	-4.597689
17 H	1.812910	-0.577412	4.319382
18 H	1.812910	-0.577412	-4.319382
19 H	-0.884546	-2.982273	2.406267
20 H	-0.884546	-2.982273	-2.406267
21 H	1.118480	-3.200619	1.732654
22 H	1.118480	-3.200619	-1.732654
23 H	2.003847	3.576366	0.000000
24 H	3.042967	2.430241	0.894687
25 H	3.042967	2.430241	-0.894687
26 H	-3.480294	2.601474	0.000000
27 H	-4.056011	1.164944	0.893306
28 H	-4.056011	1.164944	-0.893306

Table S13. Coordinates of truncated Th complex used in MCSCF calculations

1 Th	-0.219302	-0.242389	0.000000
2 C	-0.529658	2.227679	0.000000
3 C	2.242057	-2.778976	0.000000
4 C	-3.574716	-1.575980	0.000000
5 N	0.296326	0.871112	2.103983
6 N	0.296326	0.871112	-2.103983
7 O	1.542701	-1.569638	0.000000
8 O	-2.277762	-1.071438	0.000000
9 P	0.017429	2.423693	1.588553
10 P	0.017429	2.423693	-1.588553
11 Si	0.533122	0.118409	3.683064
12 Si	0.533122	0.118409	-3.683064

13 H	1.777787	0.530270	4.347954	21 H	-0.903098	2.998573	2.391449
14 H	1.777787	0.530270	-4.347954	22 H	-0.903098	2.998573	-2.391449
15 H	0.610595	-1.326902	3.426209	23 H	1.547914	-3.643336	0.000000
16 H	0.610595	-1.326902	-3.426209	24 H	2.872554	-2.865998	0.902350
17 H	-0.572272	0.340730	4.626237	25 H	2.872554	-2.865998	-0.902350
18 H	-0.572272	0.340730	-4.626237	26 H	-4.128328	-1.258401	0.901831
19 H	1.114308	3.189464	1.770072	27 H	-4.128328	-1.258401	-0.901831
20 H	1.114308	3.189464	-1.770072	28 H	-3.556936	-2.684800	0.000000

Table S14. QTAIM-calculated properties of full [M(BIPM^{TMS})(ODipp)$_2$] and truncated complexes, obtained from PBE/TZVP densities. q(M) = metal atomic charge, ρ_{BCP} = charge density at the M=C bond critical point, δ(M,C) = delocalisation index between the metal and carbon centres. All values are in atomic units

M	Structure	*q*(M)	ρ_{BCP}	*δ*(M,C)
**Ce**	Full	+2.14	0.0745	0.800
	truncated	+2.16	0.0733	0.776
**Th**	Full	+2.53	0.0752	0.624
	truncated	+2.53	0.0738	0.613
**U**	Full	+2.29	0.0853	0.788
	truncated	+2.30	0.0839	0.772

Table S15. Comparison of selected QTAIM-calculated properties of truncated complexes, obtained from RASSCF(n,2,2;11,7,11) (n = 22, 24) and CASSCF(m,m) (m = 4, 6) densities. q(M) = metal atomic charge, ρ_{BCP} = charge density at the M=C bond critical point, δ(M,C) = delocalisation index between the metal and carbon centres. All values are in atomic units.

State	*q*(M)		M-C			
			ρ_{BCP}		*δ*(M,C)	
	RAS	CAS	RAS	CAS	RAS	CAS
**Ce: ^{1}A′**	+2.84	+2.85	0.0784	0.0793	0.538	0.556
**Th: ^{1}A′**	+3.02	+3.04	0.0756	0.0757	0.493	0.492
**U: ^{3}A′**	+2.89	+2.91	0.0862	0.0867	0.532	0.537
**U: ^{3}A′′**	+2.89	+2.91	0.0859	0.0861	0.543	0.554

Table S16. Comparison of selected QTAIM-calculated properties of truncated complexes, obtained from RASSCF(n,2,2;11,7,11) (n = 22, 24) and CASSCF(m,m) (m = 4, 6) densities. ρ_{BCP} = charge density at the M=L bond critical point, δ(M,L) = delocalisation index between the metal and coordinating species centres. All values are in atomic units.

State	M-N				M-O			
	ρ_{BCP}		δ(M,N)		ρ_{BCP}		δ(M,O)	
	RAS	CAS	RAS	CAS	RAS	CAS	RAS	CAS
**Ce: ^{1}A′**	0.0717	0.0709	0.444	0.446	0.0919	0.0919	0.598	0.597
	0.0717	0.0709	0.444	0.446	0.0871	0.0871	0.581	0.581
**Th: ^{1}A′**	0.0715	0.0715	0.429	0.439	0.0933	0.0933	0.576	0.576
	0.0715	0.0715	0.429	0.439	0.0870	0.0870	0.569	0.570
**U: ^{3}A′**	0.0816	0.0813	0.473	0.478	0.1050	0.1049	0.640	0.639
	0.0816	0.0813	0.473	0.478	0.1024	0.1024	0.622	0.622
**U: ^{3}A″**	0.0798	0.0794	0.479	0.483	0.1036	0.1036	0.641	0.641
	0.0798	0.0794	0.479	0.483	0.1022	0.1023	0.622	0.620

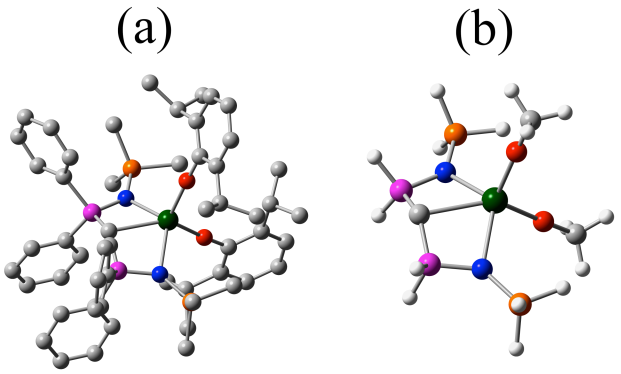

Figure S10. (a) Full M(BIPM^{TMS})(ODipp)$_2$ and (b) truncated complexes employed in quantum chemical studies. Hydrogens omitted from (a) for clarity.
